# Replacements at Structural or Functional Dimorphisms 103, 109 and 167 Distinguish HLA Class I Serologically Defined Antigens

**DOI:** 10.1111/tan.70387

**Published:** 2025-09-13

**Authors:** Kazutoyo Osoegawa, Jellina Son, Kenneth Yim, Steven G. E. Marsh, Marcelo A. Fernández Viňa

**Affiliations:** ^1^ Histocompatibility and Immunogenetics Laboratory Stanford Blood Center Palo Alto California USA; ^2^ UCL Cancer Institute Royal Free Campus London UK; ^3^ Department of Pathology Stanford University School of Medicine Palo Alto California USA

## Abstract

Amino acid variations in the HLA molecule may serve as part of serologic epitopes (residues determining epitopes: DEP) detected by allo‐antibodies. In current clinical histocompatibility practices, the presence of anti‐HLA donor‐specific antibodies (DSA) is assessed by solid phase (SP) tests with single antigen bead (SAB) panels. The antigenic risk is assessed based on the mean fluorescence intensity (MFI) values corresponding to each SAB via virtual crossmatch (VXM). To improve the accuracy of the VXM, new Associated Antigens were proposed based on DEPs; however, the antigenicity of some DEPs remained uncertain. In the current study, highly complex reactive sera were selected, and the complexity was reduced by adsorption with magnetic beads or lymphocytes followed by acid/neutralisation processes. As a proof of concept, a patient's serum was enriched with SAB coated with HLA‐A*01:01. The eluate was tested using SP‐SAB and showed reactivity with the antigens HLA‐A1, ‐A9 (excluding A2403), ‐A80, ‐B12 and ‐B76. This allowed for validation of the antibody enrichment process and identification of the serological equivalency of DEPs 167G and 167S shared by these antigens. We identified broadly reactive sera being positive with the SAB HLA‐B*35:12 (103V) and negative with the other SAB HLA‐B35 (103L). Allo‐antibodies were enriched by the adsorption/elution procedure with lymphocytes expressing HLA‐B*35:12 and with SABs coated with HLA‐B*57:01. The SAB assay using one of the eluates showed an almost identical cross‐reactivity pattern: positive with all HLA‐B SABs bearing 103V and no reactivity with SABs bearing 103L, suggesting that the enriched antibodies reacted with epitope(s) shared by HLA‐B*35:12 and HLA‐B*57:01. Additional reactivity was detected with the SABs HLA‐A32, ‐A74 and ‐Cw3. This allowed for identification of putative epitope(s) containing residues 103V and 109L. Another serum with positive reactivity to the SAB HLA‐B*35:02 showed reactivity to almost all HLA‐A SABs including ‐A*02:10 and was negative with other ‐A*02 and ‐A*69:01 SABs, suggesting that the involvement of 107G and 109F may define novel epitopes. These studies allowed us to propose 13 novel HLA‐B Associated Antigens; DEPs 103 and 109 in HLA class I were fully included in HLA Allele To Serotype (HATS) software update that allowed for a more detailed serologic characterisation of all common HLA alleles in the world.

**Abbreviations**
AA: amino acids
CIWD: common, intermediate and well‐documented
DEP: determining epitope
DSA: donor‐specific antibodies
FXM: flow cytometric crossmatch
HATS: HLA allele to serotype
IHIW: International HLA and Immunogenetics Workshops
IHWS: International Histocompatibility Workshops
IMGT: ImMunoGeneTics
IPD: ImmunoPolymorphism database
mAb: monoclonal antibody
MCS: median channel shift
MFI: mean fluorescence intensity
SAB: single antigen bead
SP: solid phase
VXM: virtual crossmatch
WD: well‐documented
WHO: World Health Organization

## 1 Introduction

The HLA genes present the highest polymorphism in the human genome; more than 40,000 HLA alleles have been registered in the IPD‐IMGT/HLA Database release version 3.58.0 [1]. Anti‐HLA allo antibodies recognise determinants resulting from amino acid (AA) differences in the distal membrane domains of HLA molecules. The immunising events leading to anti‐HLA antibody formation include pregnancies, blood transfusions as well as tissue and organ transplantation that result from the recognition of immunizer's serologic epitopes as foreign [2]. The allo‐epitopes are defined by the AA variation that distinguishes HLA proteins. In clinical Histocompatibility practices for organ and tissue transplantation, prospectively defining the anti‐HLA antibody reactivity present in recipient's serum is essential for timely assessment of donor compatibility in silico [3, 4]. These so called ‘virtual crossmatches (VXM)’ take into consideration the specificity of the patient's allo‐antibody reactivity and the donor's mismatched HLA alleles and their corresponding HLA serologically defined antigens. In order to facilitate the definition of the serological specificity corresponding to each HLA allele, HLA Allele To Serotype (HATS) program was previously developed for assigning serological specificities to each HLA allele [5]. The software development took into consideration the critical replacements at residue positions defining serological specificities. The novel Associated Antigens that were not named as the WHO recognised antigens were proposed to the WHO Nomenclature Committee for Factors of the HLA System in the most common or lowest‐digit (prototype) alleles based on the combination of the residues determining epitopes (DEP) [5]. Antigen assignments were categorised into FULL, SEROTYPE, INCOMPLETE and UNASSIGNED based on DEP matching [5, 6]. Some of the proposed antigens were validated and confirmed using the solid phase (SP) Single Antigen Bead (SAB) panel results. The validation study identified significantly different serologic reactivity patterns by the regression analyses that justified the proposed distinction as Associated Antigens for the pairs of proteins including A*66:01/A*66:02 (A‐6601/A‐6602), A*34:01/A*34:02 (A‐3401/A‐3402) that were described as being different antigens in previous IHIWS and early publications [7, 8]. The regression analyses correctly identified significantly different serologic reactivity patterns that had been identified in previous studies for the antigens corresponding to HLA‐B*51:01 and ‐B*51:02 (proposed Associated Antigen B‐5101 and Associated Antigen B5102, respectively); these proteins differ only by a single amino acid replacement at residue 171 (H/Y) that defines this residue as a DEP [5, 6, 9]. During the validation of the computationally defined antigens, the antigens that were assigned to SEROTYPE showed different serological specificities. For instance, the serological specificity of the HLA‐B*15:11 protein was defined as having the HLA‐B75 SEROTYPE [5]. However, distinctive serological reactivity was observed between SABs for HLA‐B*15:02 and ‐B*15:11; the substitution at residue 67 from Serine (S) to Tyrosine (Y) was identified as the likely determinant for the distinction between these antigens. The antigens B‐1502 and B‐1511 associated with the WHO recognised antigen HLA‐B75 were proposed by adding residue 67 as DEP for defining novel HLA‐B75 Associated Antigens in HATS v2.0 [6]. Only 22 proteins corresponding to common HLA‐B alleles had SEROTYPE level definition; these were still not proposed as FULL antigens [6], because these proteins present substitutions distinguishing them from FULL antigen category at either residue 11 or 103. In the previous studies, residue 103 (Valine vs. Leucine) was considered as DEP for the definition of distinct serotypes that were historically recognised antigen assignments B5102/B53, B4005/B50 and the possible distinction of B63 (B‐1517/B‐1516) [5, 6]; the protein HLA‐B*15:16 was a poorly identified African common variant 8W66 [10]. The eplet 103L was defined while the presence 103V was not defined as an eplet; this substitution is conserved among HLA‐A proteins and therefore, the definition of an eplet requires a substitution that is not a self‐residue at any of the HLA class I loci [11]. Of 22 proteins that had SEROTYPE assignment, 18 proteins carry substitutions at the residue 103 that is nearly dimorphic (L vs. V) except for HLA‐B*73:01 that has a distinct substitution (Methionine) at residue 103 [12]. For the ascertainment of the definition or detection of antibodies directed specific epitopes, previous work had applied methods including antibody adsorptions with lymphocytes expressing specific HLA antigens and testing the corresponding eluates in SP‐SAB assays [13]. More recently antibody adsorptions were performed with recombinant single HLA molecules immobilised on SP and testing the eluates with SP‐SAB assays and flow cytometric crossmatches (FXM) with lymphocytes expressing specific HLA antigens [14]. To assess and confirm the role of AA substitutions at residue 103 of HLA‐B as a DEP and to make finer and more precise distinctions of possible serological specificities, selected sera with complex antibody reactivity including proteins with substitution at residue 103 were processed by adsorption followed by acid elution and neutralisation. The serum adsorptions were performed with lymphocytes from subjects bearing HLA alleles corresponding to the antigens of interest [13] or magnetic beads coated with specific single HLA antigen preparations [14]. The corresponding eluates were re‐tested using the SP assays with SAB or cell‐based flow cytometric crossmatches. Here we present cases that clearly exhibited distinctive serological behaviour resulting from variation in the dimorphisms at residues 103, 109 and 167.

## 2 Materials and Methods

### 2.1 HLA Genotyping and Sample ID

HLA genotypes for all subjects were determined using the NGS HLA genotyping system (MIA FORA, Werfen Inc.). Blinded sample IDs were used for the analyses of HLA genotype and antibody profile.

### 2.2 Single Antigen Bead (SAB) Assay

LABScreen and ExPlex Single Antigen Bead (SAB) HLA class I and class II panel reagents were purchased from Thermo Fisher Scientific One Lambda Inc. Canoga Park, CA, USA. These reagents were used to perform both initial serum screening tests and for the eluate specificity assessments. In brief, serum aliquots (200 μL) were treated with 40 μL 0.5 M EDTA and adsorbed with Spherotech Polystyrene Particles as described before [15]. For each assay, a 40 μL aliquot of adsorbed serum was mixed with 6 μL of a mixture of SABs and incubated at room temperature for 30 min in the dark. After this incubation, 150 μL 1× wash buffer was added to the mixture, and beads were pelleted by centrifugation. The supernatant was removed, and the beads were washed with 1× wash buffer three more times for a total of four washes. The dried bead pellets were resuspended in diluted (1:100) Phycoerythrin (PE) conjugated Goat Anti‐Human IgG (Cat # LS‐AB2, Thermo Fisher Scientific One Lambda Inc. Canoga Park, CA, USA). The mixture was incubated at room temperature for 30 min in the dark. After the incubation, 150 μL 1× wash buffer was added to the mixture, and beads were pelleted by centrifugation. The supernatant was removed, and the beads were washed with 1× wash buffer two more times for a total of three washes. The bead pellet was resuspended in 80 μL 1× wash buffer, and signal was acquired using FLEXMAP 3D (FM3D) Flow Analyzer (Thermo Fisher Scientific One Lambda Inc. Canoga Park, CA, USA). The acquired data were imported into Fusion software (v4.4.0, Thermo Fisher Scientific One Lambda Inc. Canoga Park, CA, USA), and cut‐off values for positive and possible were adjusted. Fluorescence intensity values were imported into the mTilda database system (CareDx, HLA Data System, Houston, Texas).

### 2.3 Surveying Sera Showing Strong Positive Reactivity to One SAB and Negative Reactivity to the Other Between Two Closely Related SABs in Linear Regression Analyses

Mean fluorescence intensity (MFI) values of closely related SABs differing at DEP 103 for every subject were extracted from the database: 15,372 patients' data for HLA‐B*51:02 versus ‐B*53:01, and 3248 patients' data for HLA‐B*15:16 versus HLA‐B*15:17, ‐B*15:01 versus ‐B*15:20, ‐B*35:01 versus. ‐B*35:02, ‐B*35:01 versus ‐B*35:12, ‐B*40:02 versus ‐B*40:04, ‐B*50:01 versus ‐B*40:05, ‐B*15:03 versus ‐B*48:02, ‐B*55:01 versus ‐B*55:04, ‐B*56:01 versus ‐B*56:03. To identify a relationship between changes observed in serological reactivity in these pairs of closely related SABs, scatter plots were generated using R package ggplot2 v3.4.4 [6]. The sera with any MFI value that showed positive reactivity (MFI > 1000) were analysed with a linear regression model for every pair of SABs for HLA‐B locus antigen using the ‘lm’ function in R. Sera that showed outliers in the linear regression in the closely related two different SABs were searched; strong positive reactivity (MFI > 8000) to one SAB and negative reactivity to the other SAB (MFI < 1000). Some sera displaying distinctive reactivity with pairs of HLA‐B SAB corresponding to the same or similar serotype and differing at DEP 103 were selected for further testing and analyses. Recently, the dimorphism [Arginine (R) vs. Lysine (K)] at residue 96 of HLA‐DPB1 was described to determine additional serologic variation [16]. Scatter plots were generated for the following pairs of SABs including the same HLA‐DPA1 antigen: DPA1*02:01~DPB1*13:01 (96K) versus DPA1*02:01~DPB1*30:01 (96R), DPA1*02:01~DPB1*06:01 (96K) versus DPA1*02:01~DPB1*17:01 (96R), DPA1*01:03~DPB1*04:02 (96R) versus DPA1*01:04~DPB1*18:01 (96K). Outliers in the linear regression involving closely related SABs were searched.

### 2.4 Enrichment and Isolation of HLA Antibodies Reacting to Specific HLA Antigens Using Adsorption and Elution Processes

#### 2.4.1 Subject 1 (S1)

Subject 1 (S1) is a female recipient candidate for haematopoietic stem cell transplantation; S1's HLA genotype is shown in Table 1. The S1 serum showed strong reactivity with the SAB HLA‐A*01:01 and was negative with the SAB HLA‐A*36:01; the corresponding proteins differ only by three amino acid substitutions at residues 163, 166 and 167. To validate the specificity of the adsorption/elution process, the S1 serum was adsorbed using MagSort SAB coated with recombinant protein HLA‐A*01:01 (AE1) according to the manufacturer's protocol (Catalogue # MAG1A0101, Thermo Fisher Scientific One Lambda Inc. Canoga Park, CA, USA). The anti‐HLA specificity of the corresponding eluate was assessed by the SAB assay using LABScreen SAB standard and supplemental reagents (Catalogue #s LS1A04 and LS1AEX01, Thermo Fisher Scientific One Lambda Inc. Canoga Park, CA, USA). The SAB assay results were imported into Fusion software (v4.4.0, Thermo Fisher Scientific One Lambda Inc. Canoga Park, CA, USA).

**TABLE 1:** HLA genotypes.

ID	Genotype	Comment
S1	HLA‐A*02:17+HLA‐A*11:01^HLA‐B*18:01+HLA‐B*35:01^HLA‐C*07:01+HLA‐C*04:01^HLA‐DRB1*09:01+HLA‐DRB1*15:02^HLA‐DRB5*01:02^HLA‐DRB4*01:03^HLA‐DQA1*03:01+HLA‐DQA1*01:03^HLA‐DQB1*03:02+HLA‐DQB1*06:01^HLA‐DPA1*02:01+HLA‐DPA1*01:03+HLA‐DPB1*10:01+HLA‐DPB1*02:01	Subject carries the alleles HLA‐B*18:01+HLA‐B*35:01 shown in bold letters. A serum sample collected from this subject showed strong reactivity to the SAB HLA‐B*35:12. The proteins HLA‐B*18:01 and HLA‐B*35:01 contain residue 103V and 103L, respectively. The protein HLA‐B*35:12 carries residue 103V.
S2	HLA‐A*02:01+HLA‐A*29:02^HLA‐B*58:01+HLA‐B*56:01^HLA‐C*07:18+HLA‐C*01:02^HLA‐DRB1*08:04+HLA‐DRB1*01:01^HLA‐DQA1*01:01+HLA‐DQA1*04:01^HLA‐DQB1*04:02+HLA‐DQB1*05:01^HLA‐DPA1*01:03+HLA‐DPA1*01:03^HLA‐DPB1*03:01+HLA‐DPB1*04:02	Subject carries the alleles HLA‐B*58:01+HLA‐B*56:01 shown in bold letters. A serum sample collected from this subject showed strong reactivity to the SABs HLA‐B*57:01 and HLA‐B*56:03. The proteins HLA‐B*58:01 and ‐B*56:01 contain residue 103L, while ‐B*57:01 and ‐B*56:03 carry 103V.
S3	HLA‐A*02:01^HLA‐B*44:02 +HLA‐B*27:05^HLA‐C*05:01+HLA‐C*01:02^HLA‐DRB1*14:54+DRB1*01:01^HLA‐DRB3*02:02^HLA‐DQA1*01:04+HLA‐DQA1*01:01^HLA‐DQB1*05:03+HLA‐DQB1*05:01^HLA‐DPA1*01:03 + HLA‐DPA1*02:01+HLA‐DPB1*04:02+HLA‐DPB1*14:01	Subject is HLA‐A*02:01 homozygous shown in bold letters. The common HLA‐A2 antigens with the exception of HLA‐A210 (107G) and ‐A69 carry residue 107 W. A serum sample collected from this subject showed strong reactivity to all the HLA‐A SABs containing residue 107G, and negative for 107 W.
AE1	HLA‐A*01:01	SAB
AE2	HLA‐A*02:01^ HLA‐B*35:17 ***±*** HLA‐B*35:12 ^HLA‐C*04:01^HLA‐DRB1*14:02+HLA‐DRB1*04:07^HLA‐DRB3*01:01^HLA‐DRB4*01:03^HLA‐DQA1*05:03+HLA‐DQA1*03:01^HLA‐DQB1*03:01+HLA‐DQB1*03:02^HLA‐DPA1*01:03+HLA‐DPA1*01:03^HLA‐DPB1*04:02+HLA‐DPB1*03:01	The lymphocytes that carry the alleles HLA‐B*35:12+HLA‐B*35:17 shown in bold letters were used for enriching antibodies reacting to the antigen B‐3512 by adsorption and elution procedures. The HLA‐B*35:17 is serologically equivalent to the HLA‐B‐35:12 (B‐3512).
AE3	HLA‐B*57:01	SAB
FX1‐1	HLA‐A*02:05+HLA‐A*33:03^HLA‐B*50:01^HLA‐C*06:02^HLA‐DRB1*07:01+HLA‐DRB1*04:04^HLA‐DRB4*01:03+HLA‐DRB4*01:03^HLA‐DQA1*02:01+HLA‐DQA1*03:01^HLA‐DQB1*02:02+HLA‐DQB1*03:02^HLA‐DPA1*01:03+HLA‐DPA1*01:03^HLA‐DPB1*104:01+HLA‐DPB1*04:01	The lymphocytes that carry the allele HLA‐B*50:01 shown in bold letters were used for FXM using serum reacting to the antigen HLA‐A1 enriched by adsorption and elution procedures. The serum eluate showed negative reactivity to the SAB HLA‐B*50:01.
FX1‐2	HLA‐A*02:05+HLA‐A*02:01^HLA‐B*50:02+HLA‐B*35:01^HLA‐C*15:09+HLA‐C*06:02^HLA‐DRB1*04:04:01+HLA‐DRB1*04:06^HLA‐DRB4*01:03^HLA‐DQA1*03:01+HLA‐DQA1*03:03^HLA‐DQB1*03:02+HLA‐DQB1*04:02^HLA‐DPA1*01:03+HLA‐DPA1*01:03^HLA‐DPB1*04:02+HLA‐DPB1*02:01	The lymphocytes that carry the allele HLA‐B*50:02 shown in bold letters were used for FXM using serum reacting to the antigen HLA‐A1 enriched by adsorption and elution procedures. The serum eluate showed positive reactivity to the SABs HLA‐B*45:01 and ‐B*50:02.
FX2‐1	HLA‐A*33:03+HLA‐A*02:02^HLA‐B*35:01+ HLA‐B*15:16 ^HLA‐C*04:01+HLA‐C*14:02^HLA‐DRB1*11:02+HLA‐DRB1*03:01^HLA‐DRB3*02:02+HLA‐DRB3*02:02^HLA‐DQA1*01:05+HLA‐DQA1*05:05^HLA‐DQB1*03:19+HLA‐DQB1*05:01^HLA‐DPA1*02:01+HLA‐DPA1*01:03^HLA‐DPB1*17:01+HLA‐DPB1*02:01	The lymphocytes that carry the allele HLA‐B*15:16 shown in bold letters were used for FXM using serum reacting to the antigen B‐3512 enriched by adsorption and elution procedures. The serum eluate showed negative reactivity to the SAB HLA‐B*15:16 (103L).
FX2‐2	HLA‐A*02:01+HLA‐A*02:11^HLA‐B*35:08+ HLA‐B*15:17 ^HLA‐C*04:01+HLA‐C*07:01^HLA‐DRB1*13:02+HLA‐DRB1*11:01^HLA‐DRB3*03:01+HLA‐DRB3*02:02^HLA‐DQA1*01:02+HLA‐DQA1*05:05^HLA‐DQB1*06:04+HLA‐DQB1*03:01^HLA‐DPA1*01:03+HLA‐DPA1*01:03^HLA‐DPB1*104:01+HLA‐DPB1*02:01	The lymphocytes that carry the allele HLA‐B*15:17 shown in bold letters were used for FXM using serum reacting to the antigen B‐3512 enriched by adsorption and elution procedures. The serum eluate showed positive reactivity to the SAB HLA‐B*15:17 (103V).

*Note:* Subjects 1–3 are denoted as S1–S3. AE1 and AE3 represent MagSort SAB HLA‐A*01:01 and HLA‐B*57:01, respectively. AE2 represents lymphocytes expressing HLA‐B*35:17 + HLA‐B*35:12. FX1‐1 and FX1‐2 represent lymphocytes carrying HLA‐B*50:01 and HLA‐B*50:02, respectively. FX2‐1 and FX2‐2 represent lymphocytes carrying HLA‐B*15:16 and HLA‐B*15:17, respectively.

In addition, the S1 serum showed negative reactivity to the SABs HLA‐B*35:01, ‐B*35:02, ‐B*35:03 and ‐B*35:08 and strong reactivity to the SAB HLA‐B*35:12. This serum was used for the evaluation of reactivity possibly associated with DEP 103V. The serum was also adsorbed and eluted using lymphocytes from subject AE2 expressing the proteins HLA‐B*35:12 and HLA‐B*35:17 (Table 1). The proteins HLA‐B*35:12 and ‐B*35:17 present in subject AE2 are considered serologically equivalent since they carry identical substitutions at all DEP residues considered for the definition of HLA‐B serotypes. The adsorption and elution protocol used was modified from the previously published manuscript [13]. In brief, 440 μL serum was treated with 88 μL 0.5 M EDTA and adsorbed with Spherotech Polystyrene Particles as described previously [15]. The adsorbed serum (400 μL) was mixed with 1.0 × 10^7^ lymphocytes from subject AE2 and incubated at 21°C for 2 h. The serum‐cell suspension was centrifuged, and the adsorbed serum was stored for further testing. The recovered cell pellet was washed with 1 mL MagSort 1× Wash Buffer twice (Catalogue # MAGWABUF, Thermo Fisher Scientific One Lambda Inc. Canoga Park, CA, USA). The cell pellet was recovered by centrifugation and re‐suspended in 120 μL of MagSort Elution Buffer (Catalogue # MAGELBUF, Thermo Fisher Scientific One Lambda Inc. Canoga Park, CA, USA). The cell suspension was incubated for 1 h at 21°C on a rotator with gentle rotation. The supernatant (100 μL) was recovered by centrifugation and neutralised by adding 10 μL of MagSort Neutralisation Buffer (Catalogue # MAGNBUF, Thermo Fisher Scientific One Lambda Inc. Canoga Park, CA, USA). The same serum was also adsorbed and eluted using MagSort SAB coated with HLA‐B*57:01 (AE3) according to the manufacturer's protocol (Catalogue # MAG1B5701, Thermo Fisher Scientific One Lambda Inc. Canoga Park, CA, USA) [14]. The eluates obtained by these two independent protocols including different adsorbing antigens were tested using LABScreen SAB standard and supplemental reagents (Catalogue #s LS1A04 and LS1AEX01, Thermo Fisher Scientific One Lambda Inc. Canoga Park, CA, USA). The SAB assay results were imported into Fusion software (v4.4.0, Thermo Fisher Scientific One Lambda Inc. Canoga Park, CA, USA).

#### 2.4.2 Subject 2 (S2)

Subject 2 (S2) is a female recipient candidate included in the waiting list for a heart transplantation; S2's HLA genotype is included in Table 1. S2 carries the allele HLA‐B*56:01 and showed strong reactivity to the SAB HLA‐B*56:03 (103V) and no reactivity with the SAB for the self‐antigen HLA‐B*56:01 (103L) as expected. Adsorption/elution and eluate analyses were considered for confirmation of the role of DEP 103 in determining differential reactivity. With regard to the evaluation of self‐residues at HLA‐B for determining reactivity patterns, it should be noted that the second HLA‐B allele present in S2 is HLA‐B*58:01 (103L); therefore, S2 is homozygous 103L at HLA‐B. The S2 serum also showed negative reactivity to the SABs HLA‐B*35:01, ‐B*35:02, ‐B*35:03 and ‐B*35:08 and strong reactivity to the SAB HLA‐B*35:12; the former proteins carry 103L and the latter contains 103V, respectively. This serum was adsorbed with AE2 lymphocytes (Table 1) and the bound antibodies were eluted by acidic treatment followed by neutralisation. The same serum (S2) was also adsorbed and eluted using MagSort SAB coated with HLA‐B*57:01 (AE3). The eluates obtained from two independent protocols were also tested using LABScreen and ExPlex reagents.

### 2.5 Flow Cytometric Crossmatches

Both auto and allo T and B cell Flow Cytometric Crossmatches (FXM) were performed to evaluate the adsorbed/eluted antibody reactivity to the native HLA antigens expressed on lymphocytes. Briefly, 5 × 10^6^ lymphocytes were incubated in 1 mL Pronase solution [5 mg/mL Pronase in RPMI (Sigma cat # P‐5147), 5 mM EDTA (diluted from 0.5 M EDTA, Thermo Fisher, cat # 15575‐020) and 0.02% sodium azide] at 37°C for 15 min. At the end of the incubation, tubes containing lymphocytes were placed on ice, and immediately 200 μL cold DNase I (diluted to 100 units/mL) in Iscove's Modified Dulbecco's Medium (IMDM) was added and mixed until the visible clumps dispersed. Cold Fetal Calf Serum (FCS) (1 mL) was added and mixed by inverting the tube. Lymphocytes were collected by centrifugation, and the supernatant was removed by aspiration. The lymphocytes were resuspended in 1 mL cold FCS, and 1.0 × 10^5^ cells were used in each test. A portion of lymphocytes were stained with 7‐Aminoactinomycin D (7‐AAD: BD Pharmingen, cat # 559925) and viability was measured on FACSLyric Flow Cytometry System (BD Biosceinces). The lymphocytes (1.0 × 10^5^ cells) were pelleted by centrifugation and suspended in 30 μL serum in a 96‐well plate, and 5 μL HEPES‐saline buffer (10 mM HEPES, 0.3 M NaCl) was added. The 96‐well plate containing the samples was incubated at 21°C for 30 min. The cells were washed three times with Hanks' Balanced Salt Solution (HBSS) containing 3% bovine serum albumin (BSA) and 25 units/mL DNase I. The pre‐titrated PECy7‐CD19, PerCP‐CD3 and FITC‐antihIgG solution was added to the cells in the 96‐well plate. The plate was covered with aluminium foil and incubated at 4°C for 30 min. The cells were washed three times with HBSS containing 3% BSA and 25 units/mL DNase I to remove the unbound fluorescently labelled IgG. The cells were resuspended in 200 μL flow fixative [1% formaldehyde in Dulbecco's phosphate‐buffered saline (DPBS) without Ca^++^ and Mg^++^ (Thermo Fisher)]. The plate was read using FACSLyric Flow Cytometry System (BD Biosceinces). Median channel shift (MCS) ≥ 92 for T‐cell FXM and MCS ≥ 116 for B‐cell FXM were used for cut‐off values for positive FXM.

### 2.6 Updating HLA Allele to Serotype

HLA Allele To Serotype (HATS) program was developed in previous work, updated in the current study, and available from GitHub repository (https://github.com/kosoegawa/HATS).

## 3 Results

### 3.1 Distinctive Serological Reactivity Between SABs HLA‐B*51:02 and HLA‐B*53:01

**FIGURE 1:**
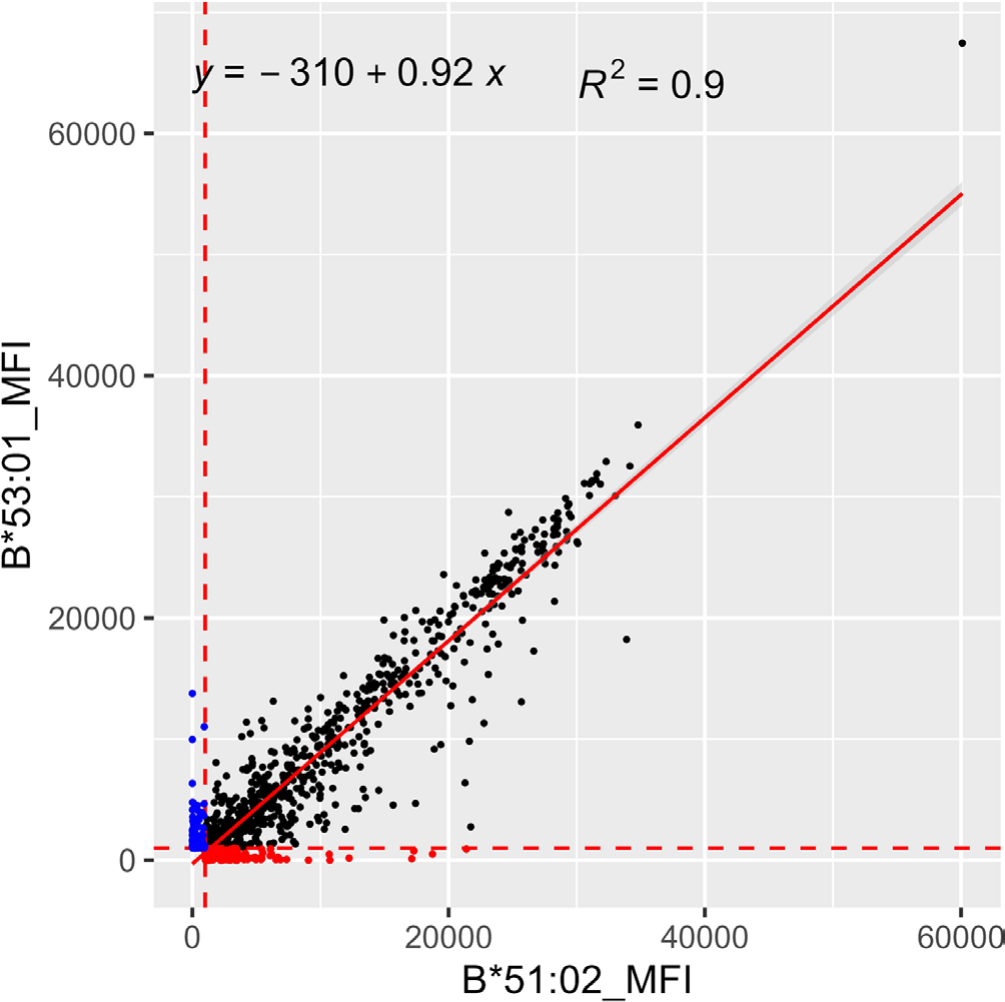
Scatter plots and linear regression analyses of the MFI values between the SABs HLA‐B*51:02 and ‐B*53:01. Scatter plots of MFI values are shown from SABs HLA‐B*51:02 and HLA‐B*53:01. The MFI values from the HLA‐B*51:02 are represented on the *X*‐axis, and HLA‐B*53:01 is on the *Y*‐axis. MFI values < 1000 on both SABs are omitted. MFI value 1000 is shown as red dotted lines on the *X* and *Y* axes. The HLA‐B*51:02 specific positive reactivities (MFI > 1000) are shown in red dots, and the HLA‐B*53:01 specific positive reactivities (MFI > 1000) are shown in blue dots. The linear regression lines are represented in a red solid line. Both SABs HLA‐B*51:02 and HLA‐B*53:01 that are in the positive range (MFI > 1000) are shown with black dots. Most black dots clustered along the linear regression line suggesting that the two antigens B5102 (HLA‐B*51:02) and B53 (HLA‐B*53:01) show strong cross‐reactivity.

The proteins HLA‐B*51:02 and ‐B*53:01 differ in residues 94, 95, 97, 103, 114, 116 and 152. Of these, the substitutions at residue 103 (103L and 103V) were considered as DEP in order to achieve distinction of the WHO recognised antigens B5102 from B53. A scatter plot was generated, and linear regression analyses were performed for the pairs of SABs HLA‐B*51:02 versus ‐B*53:01 (Figure 1). As expected, they were closely related (*R* ^2^ = 0.90), but distinctive symmetric outliers were observed, for example, strong reactivity to the SAB HLA‐B*51:02 and negative to ‐B*53:01, and vice versa (Figure 1). The scatter plot identified 21 sera that had reactivity with HLA‐B*51:02 with MFI > 5,000 and were virtually not reactive (MFI < 1000) with HLA‐B*53:01; the differential reactivity therefore can be attributed to the presence of DEP 103V. This analysis also identified 4 sera that had reactivity with B*53:01 with MFI > 5,000 and were virtually not reactive (MFI < 1000) with B*51:02; the differential reactivity therefore can be attributed to the presence of DEP 103L (Table S1A). It is interesting to note that all 4 subjects who showed strong reactivity to B53 and negative to B5102 carry HLA‐B*51:01 and/or ‐B*51:02 alleles.

### 3.2 Subject 1 (S1): Validation of Adsorption/Elution Processes

**FIGURE 2:**
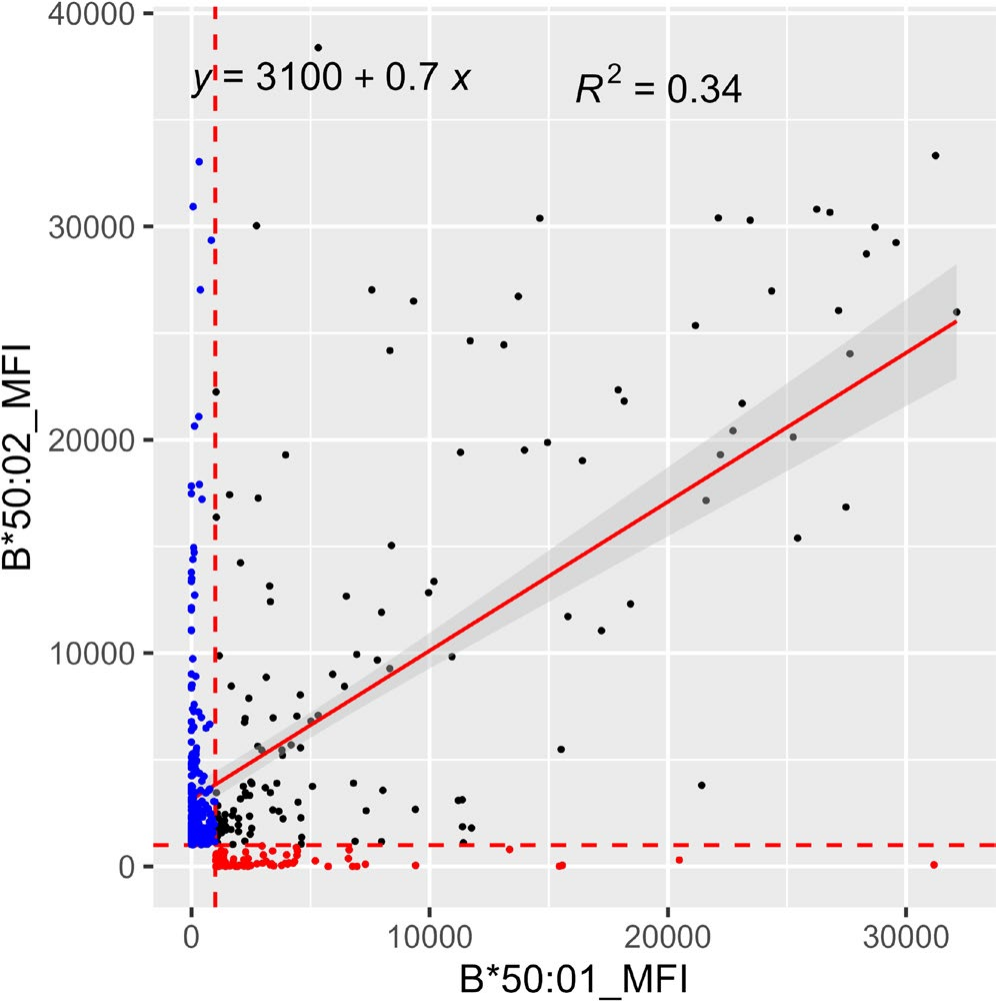
Scatter plots and linear regression analyses of the MFI values between the SABs HLA‐B*50:01 and ‐B*50:02. Scatter plots of MFI values are shown from SABs HLA‐B*50:01 and HLA‐B*50:02. The MFI values from the HLA‐B*50:01 are represented on the *X*‐axis, and HLA‐B*50:02 is on the *Y*‐axis. MFI values < 1000 on both SABs are omitted. MFI value 1000 is shown as red dotted lines on the *X* and *Y* axes. The HLA‐B*50:01 specific positive reactivities (MFI > 1000) are shown in red dots, and the HLA‐B*50:02 specific positive reactivities (MFI > 1000) are shown in blue dots. The linear regression lines are represented in a red solid line. Both SABs HLA‐B*50:01 and HLA‐B*50:02 that are in the positive range (MFI > 1000) are shown with black dots. The scatter plots suggest that the proteins HLA‐B*50:01 and ‐B*50:02 show very distinctive serological reactivity, and the single AA substitution at residue 167 is highly immunogenic.

S1 Serum showed reactivity to the SAB HLA‐A*01:01 and no reactivity to the SAB HLA‐A*36:01, and additional complex reactivities against many of HLA‐A and HLA‐B. The difference between 167G and 167W was taken into consideration for the distinction between the antigens HLA‐A1 from HLA‐A36 [5]. The ability of MagSort SAB to perform adsorption and elution of anti‐HLA antibodies was assessed using S1 serum with the MagSort SAB HLA‐A*01:01 (AE1) and SP‐SAB assays. Positive reactivity was observed to the SABs HLA‐A*01:01, ‐A*23:01, ‐A*24:02, ‐A*80:01, ‐B*15:12, ‐B*44:02, ‐B*44:03, ‐B*45:01, ‐B*50:02 and ‐B*82:01 using S1‐AE1 eluate (Table 2A). The SAB reactivity against HLA‐A*36:01, ‐A*24:03 and ‐B*50:01 was negative in this eluate (Table 2A); these proteins carry DEP 167W and differ from HLA‐A*01:01, ‐A*24:02 and ‐B*50:02 respectively. The differences in the eluate reactivity of the pairs of closely related alleles can be exclusively attributed to the presence of either 167G or 167S, compared to 167W that is present in the unreactive proteins, HLA‐A*36:01, ‐A*24:03 and ‐B*50:01 with this eluate. To evaluate the presence of at least one or more epitopes in HLA molecules expressed on lymphocytes in the native configuration, T cell FXMs were performed using S1‐AE1 eluate against FX1‐1 cells that contain HLA‐B*50:01 homozygous, and FX1‐2 cells that carry HLA‐B*50:02 and HLA‐B*35:01 (Table 1). The HLA‐B*50:01 and HLA‐B*50:02 proteins differ by the single amino acid replacement noted at residue 167 (167W and 167S, respectively). Linear regression model analysis showed a significant difference in serological reactivity between HLA‐B*50:01 and HLA‐B*50:02 using MFI values from SP‐SAB data with 3248 sera, *R* ^2^ = 0.34 (Figure 2, Table S1B). The FX1‐1 expresses the serologic antigen HLA‐B50 represented by HLA‐B*50:01, and the FX1‐2 expresses the HLA‐B*50:02 corresponding to the antigen HLA‐B45. The T cell FXM was negative with FX1‐1 cells while it was positive with FX1‐2 cells. These results were concordant with the results obtained by SP‐SAB assays and indicate that the untreated serum S1 and the eluate S1‐AE1 detect epitopes detected in the SAB assay and that are also expressed on the cell surface of lymphocytes (Table 2B).

**TABLE 2A:** SAB assay using untreated S1 serum and its eluate prepared by adsorption/elution using the antigen HLA‐A1 (AE1).

Broad antigen	Split	Associated	SAB	Untreated	AE1 (A*01:01)	Score	167
A1			A*01:01	7856	4368	POS	G
			A*01:02	5936	3928	POS	G
A3		A‐0301	A*03:01	30531	31	NEG	W
			A*03:02	27724	0	NEG	W
A9	A23	A‐2301	A*23:01	31664	3603	POS	G
	A24	A‐2402	A*24:02	26807	3015	POS	G
		A2403	A*24:03	31744	0	NEG	W
A36			A*36:01	238	15	NEG	W
A80			A*80:01	3413	1147	POS	G
B12	B44	B‐4402	B*44:02	29814	4801	POS	S
			B*44:03	30712	4941	POS	S
	B45		B*45:01	29490	4945	POS	S
			B*50:02	27118	4486	POS	S
B21	B49		B*49:01	30920	36	NEG	W
	B50		B*50:01	28965	71	NEG	W
		B4005	B*40:05	31437	52	NEG	W
B15	B76		B*15:12	28513	5684	POS	G
			B*15:14	NA	NA	NA	S
B82			B*82:01	30569	4462	POS	S

*Note:* SAB assays were performed using serum collected from S1 and its eluate prepared by adsorption/elution using the antigen HLA‐A1 (AE1, Table 1). ‘Broad Antigen’, ‘Split’ and ‘Associated’ columns show WHO recognised antigens and previously proposed antigens (with dash). ‘SAB’ column shows HLA proteins coated on SABs. ‘Untreated’ column shows MFI values using untreated serum S1. ‘AE1 (A*01:01)’ column shows MFI values using eluate using the SAB HLA‐A*01:01 (Table 1, AE1). ‘Score’ column shows ‘POS(ITIVE)’ or ‘NEG(ATIVE)’ based on MFI values in ‘AE1’ column (cut off value: 1000). ‘167’ column shows amino acid residues at position 167: Glycine (G), Tryptophan (W) or Serine (S). Positive reactivity was observed to the antigen containing DEP 167G/S suggesting that these AA are equivalent at this position in determining serologic epitope, while negative reactivity was observed to the antigen containing DEP 167W.

**TABLE 2B:** T cell FXM for confirming cross‐reactivity between antigens A1 and B45.

ID	Serum		Cell FX1‐1	Cell FX1‐2
	MFI to SAB		B*50:01+B*50:01	B*35:01+B*50:02
	B*50:01	B*50:02	T‐MCS	T‐MCS
AE1	71	4486	0 (NEG)	104 (POS)

*Note:* Table shows T‐FXM results. ‘Serum’ column shows MFI values to the SABs HLA‐B*50:01 and HLA‐B*50:02 (Table 2A), and ‘Cell FX1‐1’ and ‘Cell FX1‐2’ columns show HLA‐B genotypes for the cells used (Table 1). FX1‐1 cell expresses HLA‐B*50:01 (homozygous) and FX1‐2 cell expresses HLA‐B*50:02 shown in bold letters. ‘POS’ indicates positive (MCS ≥ 92), and ‘NEG’ indicates negative (MCS < 92). Eluate S1‐AE1 was obtained from subject 1 (S1) adsorbed with SAB HLA‐A*01:01 (Table 1, AE1). The proteins HLA‐A*01:01, ‐B*50:01 and ‐B*50:02 carry 167G, 167W and 167S, respectively; The T‐FXM shows positive to the cells expressing HLA‐B*50:02 (167S), while negative to the cells expressing HLA‐B*50:01 (homozygous, 167W), being consistent with the results from Table 2A. The results confirmed that the serum eluted using the recombinant protein HLA‐A*01:01 containing DEP 167G shows positive reactivity to the HLA‐B*50:02 containing DEP 167S, but negative to the HLA‐B*50:01 containing DEP 167W.

### 3.3 Subject 2 (S2): Identification of Anti‐HLA Antibodies Directed to Discontinuous DEPs 103V + 109L Shared by Alleles at HLA‐A, B and C Loci

**FIGURE 3:**
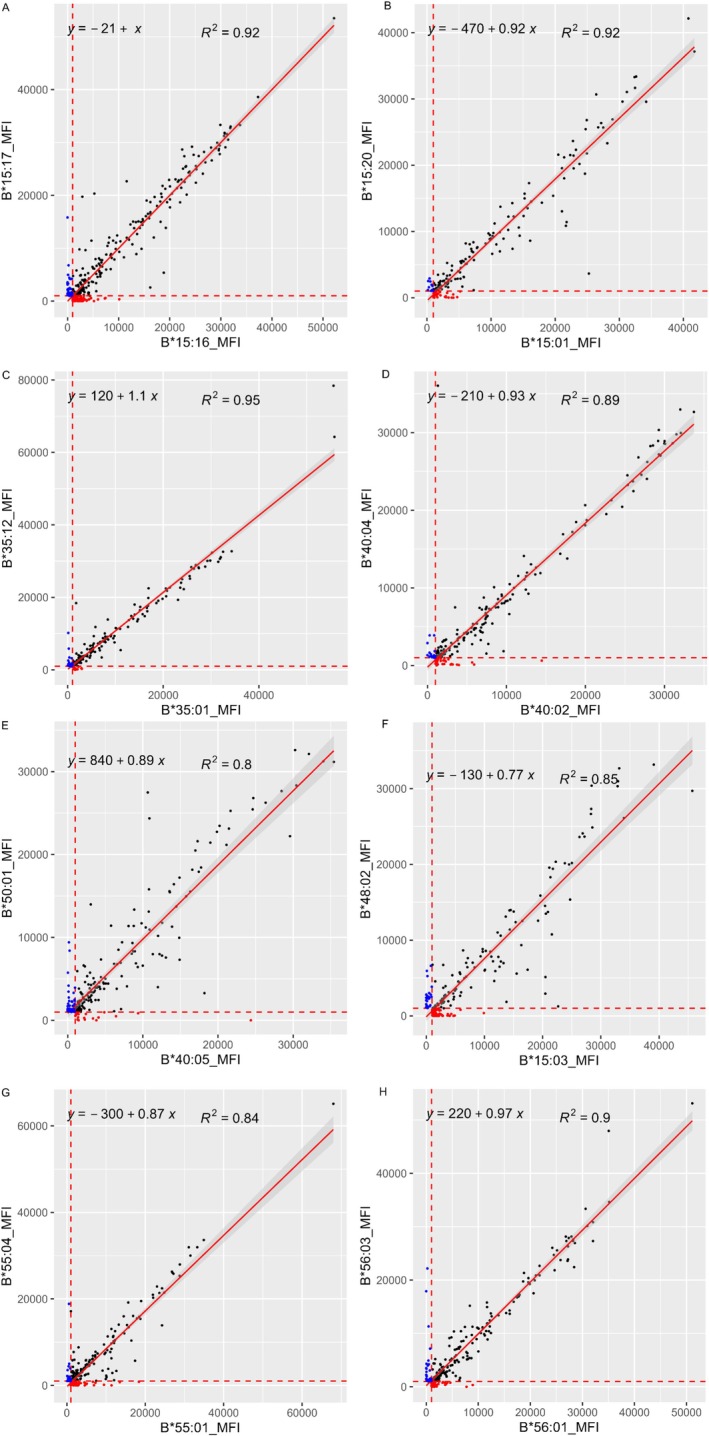
Scatter plots and linear regression analyses of the MFI values between the closely related SABs differing at DEP 103. Scatter plots of MFI values are shown between the closely related SABs differing at DEP 103. The MFI values from the SABs HLA‐B*15:16, B*15:01, B*35:01, B*40:02, B*40:05, B*15:03, B*55:01, and B*56:01 are represented on the *X*‐axis, and HLA‐B*15:17, B*15:20, B*35:12, B*40:04, B*50:01, B*48:02, B*55:04, and B*56:03 are on the *Y*‐axis. MFI values < 1000 on both SABs are omitted. MFI value 1000 is shown as red dotted lines on the *X* and *Y* axes. The SABs HLA‐B*15:16, B*15:01, B*35:01, B*40:02, B*40:05, B*15:03, B*55:01 and B*56:01 specific positive reactivities (MFI > 1000) are shown in red dots, and the HLA‐B*15:17, B*15:20, B*35:12, B*40:04, B*50:01, B*48:02, B*55:04 and B*56:03 specific positive reactivities (MFI > 1000) are shown in blue dots. The linear regression lines are represented as a red solid line. Both SABs that are in the positive range (MFI > 1000) are shown with black dots. Most black dots clustered along the linear regression lines, suggesting that these two closely related antigens show strong cross‐reactivity.

**FIGURE 4:**
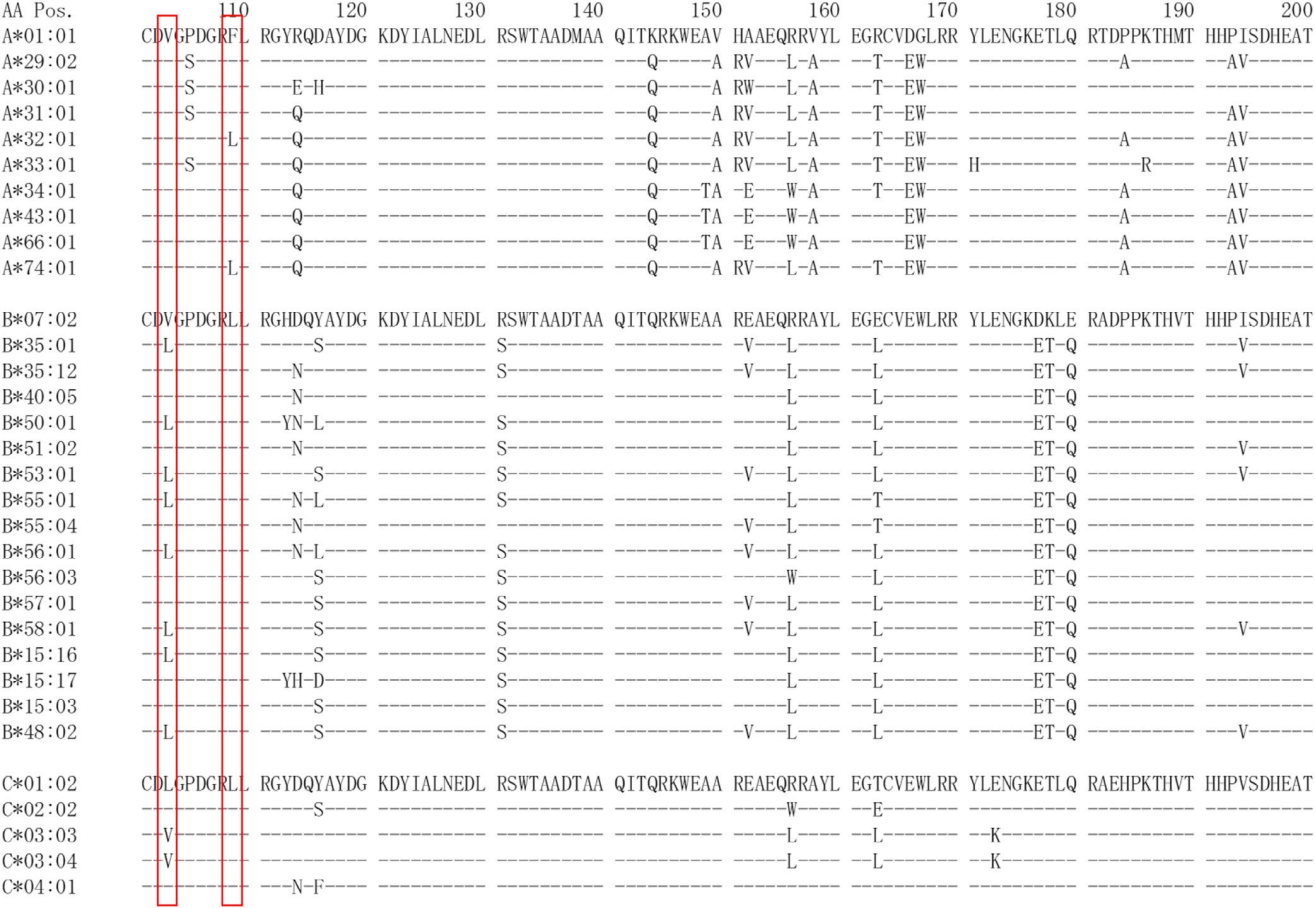
AA sequence alignments for HLA‐A, ‐B and ‐C. The HLA‐A, HLA‐B and HLA‐C AA sequence alignments between residues 101 and 200 were shown. The broad antigen HLA‐Cw3 contains DEP103V that is unique among HLA‐C antigens; the other HLA‐C antigen contains 103L (bottom). The DEP 103V can be observed in all HLA‐A antigens; therefore, the DEP103V alone cannot explain the reactivity to the SABs HLA‐A*32:01 and HLA‐A*74:01. The residue 109L is recognised as unique in the HLA‐A*32:01 and HLA‐A*74:01, while the other HLA‐A antigen contains 109F (top). The residue 109L is recognised for almost all HLA‐B and HLA‐C antigens.

The SP‐SAB test with S2 serum showed broad reactivity, indicating a profile corresponding to a highly sensitised patient; S2 serum showed strong reactivity to the SABs HLA‐B*15:03, ‐B*15:17, ‐B*35:12, ‐B*40:05, ‐B*51:02, ‐B*55:04, ‐B*56:03 and ‐B*57:01, while it showed negative or weak reactivity to ‐B*48:02, ‐B*15:16, ‐B*35:01, ‐B*50:01, ‐B*53:01, ‐B*55:01, ‐B*56:01 and ‐B*58:01 (Table 3A). The former and latter groups contain residues 103V and 103L, respectively. The two‐field allele HLA genotype from of S2 is shown in Table 1. The scatter plots and linear regression analyses between closely related SABs differing at DEP 103 are shown in Figure 3. The proteins HLA‐B*35:12, ‐B*55:04, ‐B*56:03 and ‐B*48:02 were reported as SEROTYPE assignments due to the difference at residue 103 in the previous manuscript. From the observation of differential reactivity in serum S2, we speculated that the strong reactivity to the SABs HLA‐B*15:03, ‐B*15:17, ‐B*35:12, ‐B*40:05, ‐B*51:02, ‐B*55:04, ‐B*56:03 and ‐B*57:01 could involve epitope(s) defined by the presence of DEP 103V. In order to confirm the presence of shared epitope(s) related to residue 103V, serum S2 was adsorbed with lymphocytes carrying HLA‐B*35:12+HLA‐B*35:17 (AE2) and with the MagSort beads coated with the recombinant protein HLA‐B*57:01 (AE3). The bound antibodies were eluted from either lymphocytes or SAB; the serum eluates were re‐tested using the SAB assay. The analyses of both eluates showed almost identical reactivity patterns recovered from adsorptions with two different antigens (B‐3512 vs. B57) (Figure S1). These results strongly suggest the presence of antibodies reacting to one or more epitopes shared by proteins carrying DEP 103V (Table 3A). All HLA‐B SABs containing DEP 103V showed positive reactivity, while the HLA‐B SABs containing 103L were all negative (Table 3A). In addition to the reactivity related to the HLA‐B SABs containing DEP 103V, the sera showed strong reactivity with the SABs HLA‐C*03:02, HLA‐C*03:03 and HLA‐C*03:04. The common alleles corresponding to the serologic antigen HLA‐Cw3 carry DEP 103V that is unique among common HLA‐C antigens; the other HLA‐C antigens carry 103L (Figure 4). In addition to the HLA‐B and C reactivity, these eluates also showed reactivity with the SAB coated with HLA‐A*32:01 and HLA‐A*74:01; and the residue 103V is present in all common HLA‐A proteins, including those present in subject S2; therefore, the DEP 103V alone cannot explain the reactivity to the SABs HLA‐A*32:01 and HLA‐A*74:01 (Figure 4). To understand HLA‐A reactivity with the S2‐AE2 eluate, we conducted further examination, attempting to identify any unique residue present in both HLA‐A*32:01 and HLA‐A*74:01 and absent in HLA‐A proteins with antigens different from HLA‐A32 and ‐A74. It became readily apparent that residue 109L could determine part of epitope(s) detected in S2‐AE2 and S2‐AE3 eluates. Residue 109L is uniquely present in the alleles HLA‐A*32:01 and HLA‐A*74:01, while the other common HLA‐A proteins with serologic antigens other than HLA‐A32 or A74 carry 109F (Figure 4).

**TABLE 3A:** SAB assay using sera prepared from S2 serum.

Broad	Split	Associated	SAB	Untreated	AE2	AE3	103	109
A2		A‐0201	A*02:01	0	396	6	V	F
A9	A23	A‐2301	A*23:01	32914	0	0	V	F
	A24	A‐2402	A*24:02	33320	0	0	V	F
		A2403	A*24:03	34655	0	0	V	F
A19	A29	A‐2902	A*29:02	73	0	0	V	F
	A32	A‐3201	A*32:01	20487	19750	12119	V	L
	A74		A*74:01	12951	19410	11633	V	L
B7			B*07:02	31339	7054	2738	V	L
			B*07:14	31556	7182	2774	V	L
B8			B*08:01	10431	7505	4057	V	L
B13			B*13:01	23010	3796	628	L	L
			B*13:02	27701	4770	820	L	L
B14	B64		B*14:01	18889	16808	10982	V	L
	B65		B*14:02	16066	15669	9729	V	L
B15	B62	B‐1501	B*15:01	21152	19126	10799	V	L
			B*15:04	17488	17671	9877	V	L
			B*15:06	19051	17816	10183	V	L
			B*15:07	20242	18520	10720	V	L
			B*15:27	19786	18267	10147	V	L
		B‐1520	B*15:20	7335	3803	1076	L	L
		B‐1524	B*15:24	16378	16155	9317	V	L
	B63	B‐1516	B*15:16	3123	0	954	L	L
		B‐1517	B*15:17	19391	18475	11696	V	L
	B75	B‐1502	B*15:02	18578	17597	10080	V	L
			B*15:21	18755	17368	10185	V	L
		B‐1511	B*15:11	15397	14942	9203	V	L
	B76		B*15:12	26366	16772	8859	V	L
	B77		B*15:13	14707	14036	8477	V	L
B15/ B70	B71	B‐1510	B*15:10	19611	18090	10932	V	L
			B*15:18	19340	18077	10803	V	L
	B72	B‐1503	B*15:03	19776	18435	10757	V	L
		B‐4802	B*48:02	1756	1316	3	L	L
B18			B*18:01	20142	19233	12415	V	L
B27		B‐2705	B*27:04	30655	18563	11048	V	L
			B*27:05	29844	19153	11585	V	L
			B*27:06	29375	18139	11140	V	L
		B2708	B*27:08	30617	19447	11726	V	L
B35		B‐3501	B*35:01	1108	965	4	L	L
			B*35:03	718	660	0	L	L
			B*35:08	720	755	0	L	L
		B‐3502	B*35:02	662	590	0	L	F
		B‐3512	B*35:12	17358	17805	10458	V	L
B37		B‐3701	B*37:01	18669	18742	11300	V	L
B16	B38	B‐3801	B*38:01	16825	17716	11027	V	L
			B*38:02	19502	19553	11593	V	L
	B39	B3901	B*39:01	19218	18217	11584	V	L
			B*39:04	18247	17587	11189	V	L
			B*39:05	18898	18646	11509	V	L
			B*39:06	16425	16454	9940	V	L
		B3902	B*39:02	20308	19544	11775	V	L
			B*39:13	21171	20528	12325	V	L
B40	B60	B‐4001	B*40:01	27892	7626	2709	V	L
	B61	B‐4002	B*40:02	27481	18082	11213	V	L
			B*40:03	27448	18051	10474	V	L
			B*40:06	26079	16977	10015	V	L
		B‐4004	B*40:04	27101	2317	0	L	L
B41			B*41:01	7854	5152	1641	V	L
			B*41:02	9031	5806	2139	V	L
B42			B*42:01	10290	4813	2280	V	L
			B*42:02	9267	4618	2022	V	L
B12	B44	B‐4402	B*44:02	18719	16669	9939	V	L
			B*44:03	19985	17594	10295	V	L
	B45		B*45:01	13012	1633	0	L	L
			B*50:02	12937	1596	0	L	L
B46			B*46:01	17987	18791	11849	V	L
B47		B‐4701	B*47:01	20863	13706	8613	V	L
B48		B‐4801	B*48:01	27099	8064	3912	V	L
B21	B49		B*49:01	3304	1788	0	L	L
	B50		B*50:01	2230	1376	0	L	L
		B4005	B*40:05	16702	17517	10552	V	L
B5	B51	B‐5101	B*51:01	15408	15819	9492	V	L
		B5102	B*51:02	15985	16968	10114	V	L
	B52		B*52:01	13260	14526	8497	V	L
B53			B*53:01	727	694	0	L	L
B22	B54		B*54:01	17	0	0	L	L
	B55	B‐5501	B*55:01	1298	0	0	L	L
			B*55:02	38	0	0	L	L
		B‐5504	B*55:04	17286	17427	10456	V	L
	B56	B‐5601	B*56:01	0	0	0	L	L
		B‐5603	B*56:03	16789	16864	9760	V	L
B17	B57		B*57:01	20174	19483	12609	V	L
			B*57:03	18562	18182	11668	V	L
	B58		B*58:01	0	0	0	L	L
B59			B*59:01	81	0	0	L	L
B67		B‐6701	B*67:01	21905	20313	12337	V	L
B73			B*73:01	28662	307	0	M	L
B78		B‐7801	B*78:01	15441	16051	9556	V	L
B81			B*81:01	29686	7013	2980	V	L
B82			B*82:01	12417	0	0	L	L
Cw1			C*01:02	38	0	200	L	L
Cw3	Cw9		C*03:03	22001	16915	9561	V	L
	Cw10	C‐0304	C*03:02	19755	15003	8683	V	L
			C*03:04	20997	15620	8834	V	L
Cw7		C‐0701	C*07:01	162	0	0	L	L

*Note:* SAB assays were performed using untreated serum, 2 eluates from S2 (Table 1). The eluates were prepared by adsorption and elution using cells expressing HLA‐B*35:12+HLA‐B*35:17 (Table 1 AE2) and using MagSort SAB HLA‐B*57:01 (Table 1, AE3). ‘Broad’, ‘Split’ and ‘Associated’ columns show WHO recognised antigens and proposed antigens (with dash). ‘SAB’ column shows HLA proteins coated on SABs. ‘Untreated’ column shows MFI values using the untreated serum from subject 2. ‘AE2 (B*35:12 + B*35:17)’ column shows MFI values using serum eluted using lymphocytes expressing HLA‐B*35:12+HLA‐B*35:17 (AE2). ‘AE3 (B*57:01)’ column shows MFI values using serum eluted using Magsort SAB HLA‐B*57:01 (AE3). ‘103’ and ‘109’ column shows AA residues at these positions; valine (V), leucine (L) or methionine (M) at residue 103, phenylalanine (F) or leucine (L) at residue 109. Self‐proteins (HLA‐A*02:01, ‐A*29:02, ‐B*56:01, ‐B*58:01, ‐C*01:02 and C*07:01) were shown in bold letter; HLA‐C*07:18 is serologically equivalent to HLA‐C*07:01. Only residues 103V + 109L combination showed strong positive reactivity, while the other combination (103L + 109L, 103L + 109F or 103M + 109L) showed negative reactivity (grey highlighted).

The reactivity identified in the eluate (S2‐AE2) recovered after the adsorption with lymphocytes expressing HLA‐B*35:12+HLA‐B*35:17 also showed reactivity with the SAB HLA‐B*15:17 (103V + 109L), no reactivity with HLA‐B*15:16 (103L + 109L) using the SAB assay. The proteins HLA‐B*15:17 and HLA‐B*15:16 are the prototypes for the proposed Associated Antigens rooted to the WHO recognised antigen HLA‐B63. T cell FXM were performed using lymphocytes expressing HLA‐B*35:01+HLA‐B*15:16 (FX2‐1 in Table 1) and HLA‐B*35:08+HLA‐B*15:17 (FX2‐2 in Table 1) and not bearing any other HLA class I allele that could be associated with possible additional reactivity. The T FXM using FX2‐1 lymphocytes was negative while T FXM using FX2‐2 lymphocytes was positive confirming that the alloreactive anti‐HLA antibodies adsorbed and eluted from the antigen corresponding to HLA‐B*35:12 and ‐B*35:17 (here named B‐3512 carries 103V + 109L) were reactive with lymphocytes expressing HLA‐B*15:17 (103V + 109L) and were negative with the lymphocytes expressing HLA‐B*15:16 (103L + 109L) (Table 3B).

**TABLE 3B:** T FXM for confirming the distinctive antigens B‐1516 and B‐1517 associated to the antigen HLA‐B63.

Serum ID	MFI to SAB		Cell FX2‐1	Cell FX2‐2
			B*35:01+B*15:16	B*35:08+B*15:17
	B*15:16	B*15:17	T‐MCS	T‐MCS
S2‐AE2	0	18475	45 (NEG)	334 (POS)
B (Bw4 positive)	10547	NA	265 (POS)	253 (POS)

*Note:* Table shows T‐FXM results. ‘MFI to SAB’ column shows MFI values to the SABs HLA‐B*15:16 and HLA‐B*15:17, and ‘Cell FX2‐1’ and ‘Cell FX2‐2’ columns show HLA‐B genotypes for the cells used (Table 1). FX2‐1 cell expresses HLA‐B*15:16 and FX2‐2 cell expresses HLA‐B*15:17 shown in bold letters. ‘POS’ indicates positive (MCS ≥ 92), and ‘NEG’ indicates negative (MCS < 92). ‘S2‐AE2’ was prepared by adsorption and elution from serum obtained from S2 (Table 1) using lymphocytes expressing HLA‐B*35:12+HLA‐B*35:17 (AE2: Table 3A). Serum B was recognised as Bw4 reactive using standard SAB panel that lacks the SAB HLA‐B*15:17 was used as positive control to show positive FXM results for both cells.

### 3.4 Confirmation of Valine 103 as Being a DEP Residue

The SAB assay using serum collected from S1 showed highly sensitised status including strong reactivity to the SAB HLA‐B*35:12 and weak or negative reactivity to the SABs HLA‐B*35:01, ‐B*35:02, ‐B*35:03 and ‐B*35:08 (Table 4). The residue 103V is likely involved in the differential reactivity among the broad antigen HLA‐B35 as observed in S2 serum. However, the serum did not show the full DEP 103V related reactivity pattern after adsorption and elution using AE2 cells. Contrasting with S2, the HLA‐B proteins expressed on S1 cells include HLA‐B*18:01 and HLA‐B*35:01 that carry 103V and 103L, respectively (Table 4); most likely the short 103V pattern resulted from induction of tolerance to the repertoire of self‐epitopes in the HLA‐B*18:01. This partial reactivity pattern with 103V + 109L bearing proteins suggests that additional DEPs are likely to be involved in determining the HLA epitope(s) detected by the S1‐AE2 eluate. S1 serum was also adsorbed with lymphocytes carrying HLA‐B*35:12 + HLA‐B*35:17 (AE2 in Table 1) and with the MagSort beads coated with the HLA‐B*57:01 (AE3 in Table 1). The S1‐AE2 and S1‐AE3 eluates corresponding to adsorptions from cells and the beads bearing different HLA‐B antigens were re‐tested using the SAB assay. Unlike S2‐AE2 eluates, the S1‐AE2 eluate obtained after adsorption with AE2 (cells bearing HLA‐B*35:12 and ‐B*35:17, both corresponding to the antigen B‐3512) showed reactivity with several but not all HLA‐B alleles bearing 103V + 109L (highlighted in Table 4): no reactivity with self HLA‐B*18:01 as well as low or absent reactivity with ‐B*14:01, ‐B*14:02, ‐B*44:02, ‐B*44:03, and many of the ‐B*15 and ‐B*51 proteins. The S1‐AE2 eluate did not show reactivity with the SABs bearing HLA‐A*32:01, HLA‐A*74:01, HLA‐C*03:02, HLA‐C*03:03 and HLA‐C*03:04. The comparison of the eluates corresponding to these sera from S1 and S2 may lead to the theory that a combination of DEPs forms epitope(s) that may sometimes include non‐polymorphic residues. The S1‐AE2 eluate showed additional reactivity with SAB HLA‐B*40:04, ‐B*13:01 and ‐B*13:02; these proteins carry 103L + 109L (Table 4). This additional reactivity suggests the detection of additional complex epitopes shared by these proteins with HLA‐B*35:12 and/or ‐B*35:17 present in the adsorption cells (Table 4). The S1‐AE3 eluate obtained after adsorption with AE3 (SAB HLA‐B*57:01) showed reactivity with the same HLA‐B proteins bearing 103V that were positive with S1‐AE2 eluate. In addition, this eluate showed reactivity with HLA‐A and B proteins bearing DEP 82‐83 LR associated with Bw4. The results obtained from the S1‐AE3 eluate indicate that S1 serum contains at least two sets of allo‐antibodies directed to different HLA‐B epitopes present in the protein HLA‐B*57:01, one determined by DEP 103V and the other determined by the presence of DEP 82‐83 LR. A third set of antibody reactivity with additional proteins may be directed to a less characterised cross‐reactive group including antigens associated with HLA‐B7 + ‐B22 + ‐B17 + ‐B63 related to the presence of the amino acid substitutions at residues 69 and 71 of HLA‐B; therefore, it appears that the S1‐AE3 eluate detects reactivity with some proteins having DEPs 103L and 109L.

**TABLE 4:** SAB assay using sera prepared from S1 serum.

Broad	Split	Associated	SAB	Untreated	AE2	AE3	69	82	83	103	109
A2			A*02:01	118	100	49				V	F
A9	A23	A‐2301	A*23:01	33496	0	22029		L	R	V	F
	A24	A‐2402	A*24:02	29077	0	17820		L	R	V	F
		A2403	A*24:03	32649	0	22143		L	R	V	F
A10	A25		A*25:01	33544	0	19147		L	R	V	F
A11			A*11:01	337	17	92				V	F
A19	A32	A‐3201	A*32:01	33504	537	23851		L	R	V	L
	A74		A*74:01	21716	576	2561				V	L
B7			B*07:02	30302	18093	27685	A			V	L
			B*07:14	30652	16069	26885	A			V	L
B8			B*08:01	35397	17366	15722				V	L
B13			B*13:01	29090	3287	8806		L	R	L	L
			B*13:02	33741	5443	12592		L	R	L	L
B14	B64		B*14:01	16018	298	813				V	L
	B65		B*14:02	14448	578	424				V	L
B15	B62	B‐1501	B*15:01	32521	1051	8667				V	L
			B*15:04	28283	1979	7758				V	L
			B*15:06	27969	77	7344				V	L
			B*15:07	30670	2971	8479				V	L
			B*15:27	30562	354	7187				V	L
		B‐1520	B*15:20	26767	18	6670				L	L
		B‐1524	B*15:24	25333	773	8543		L	R	V	L
	B63	B‐1516	B*15:16	31940	0	22916	A	L	R	L	L
		B‐1517	B*15:17	34030	732	23497	A	L	R	V	L
	B75	B‐1502	B*15:02	16291	0	6822				V	L
			B*15:21	16739	0	6638				V	L
		B‐1511	B*15:11	6742	70	1961				V	L
	B76		B*15:12	31142	507	6796				V	L
	B77		B*15:13	14886	0	6916		L	R	V	L
B15/ B70	B71	B‐1510	B*15:10	28330	1803	801				V	L
			B*15:18	25291	394	246				V	L
	B72	B‐1503	B*15:03	27773	1773	4752				V	L
		B‐4802	B*48:02	20489	0	950				L	L
B18			B*18:01	3	0	0				V	L
B27		B‐2705	B*27:04	32985	7153	24698	A	L	R	V	L
			B*27:05	32284	6994	24873	A	L	R	V	L
			B*27:06	33498	7220	24499	A	L	R	V	L
		B2708	B*27:08	32079	7805	26781	A			V	L
B35		B‐3501	B*35:01	108	0	77				L	L
			B*35:03	0	0	15				L	L
			B*35:08	0	0	18				L	L
		B‐3502	B*35:02	51	0	0				L	F
		B‐3512	B*35:12	22504	6851	1796				V	L
B37		B‐3701	B*37:01	25553	1299	4687		L	R	V	L
B16	B38	B‐3801	B*38:01	32166	4992	6075		L	R	V	L
			B*38:02	34647	6344	7267		L	R	V	L
	B39	B3901	B*39:01	30837	4619	2614				V	L
			B*39:04	30550	3518	1642				V	L
			B*39:05	32537	5497	1965				V	L
			B*39:06	25110	2192	1361				V	L
		B3902	B*39:02	30755	5374	2574				V	L
			B*39:13	31688	6277	1924				V	L
B40	B60	B‐4001	B*40:01	32618	17634	16874				V	L
	B61	B‐4002	B*40:02	33963	10715	10347				V	L
			B*40:03	33470	9815	9508				V	L
			B*40:06	33840	9301	8605				V	L
		B‐4004	B*40:04	33332	5394	6437				L	L
B41			B*41:01	34777	14571	13564				V	L
			B*41:02	34370	14535	12426				V	L
B42			B*42:01	31921	15963	27021	A			V	L
			B*42:02	33714	14787	25614	A			V	L
B12	B44	B‐4402	B*44:02	32668	81	3995		L	R	V	L
			B*44:03	33400	73	4760		L	R	V	L
	B45		B*45:01	31859	0	48				L	L
			B*50:02	29751	0	0				L	L
B46			B*46:01	16522	1379	4495				V	L
B47		B‐4701	B*47:01	31055	2798	5672		L	R	V	L
B48		B‐4801	B*48:01	33907	15911	16708				V	L
B21	B49		B*49:01	33477	68	3202		L	R	L	L
	B50		B*50:01	31249	0	139				L	L
		B4005	B*40:05	34004	6217	1826				V	L
B5	B51	B‐5101	B*51:01	13172	1339	2425		L	R	V	L
		B5102	B*51:02	12220	1323	2567		L	R	V	L
	B52		B*52:01	18341	1289	2311		L	R	V	L
B53			B*53:01	14208	0	4252		L	R	L	L
B22	B54		B*54:01	30883	72	19360	A			L	L
	B55	B‐5501	B*55:01	32873	390	20954	A			L	L
			B*55:02	33357	300	21027	A			L	L
		B‐5504	B*55:04	32535	7479	22302	A			V	L
	B56	B‐5601	B*56:01	34383	273	21268	A			L	L
		B‐5603	B*56:03	33950	2206	21157	A			V	L
B17	B57		B*57:01	33283	5910	28271	A	L	R	V	L
			B*57:03	34090	8451	26919	A	L	R	V	L
	B58		B*58:01	28575	0	22878	A	L	R	L	L
B59			B*59:01	26852	0	4903		L	R	L	L
B67		B‐6701	B*67:01	31270	7467	26516	A	L	R	V	L
B73			B*73:01	33134	4159	14909	A			M	L
B78		B‐7801	B*78:01	11712	1525	963				V	L
B81			B*81:01	30723	17426	26107	A			V	L
B82			B*82:01	32262	0	18753	A			L	L
Cw3	Cw9		C*03:03	26273	29	965				V	L
	Cw10	C‐0304	C*03:02	14637	0	538				V	L
			C*03:04	19173	0	787				V	L
Cw4		C‐0401	C*04:01	339	0	233				L	L
Cw7		C‐0701	C*07:01	2713	0	469				L	L

*Note:* SAB assays were performed using untreated serum, 2 eluates from S1 (Table 1). The eluates were prepared by adsorption and elution using cells expressing HLA‐B*35:12 + HLA‐B*35:17 (Table 1 AE2) and using MagSort SAB HLA‐B*57:01 (Table 1, AE3). ‘Broad’, ‘Split’ and ‘Associated’ columns show WHO recognised antigens and proposed antigens (with dash). ‘SAB’ column shows HLA proteins coated on SABs. ‘Untreated’ column shows MFI values using the untreated serum from S1. ‘AE2’ column shows MFI values using eluate with lymphocytes expressing HLA‐B*35:12 + HLA‐B*35:17. ‘AE3’ column shows MFI values using eluate with Magsort SAB HLA‐B*57:01. ‘69’, ‘82’, ‘83’, ‘103’ and ‘109’ column shows amino acid residues at these positions; Valine (V) or Leucine (L) at residue 103, Phenylalanine (F) or Leucine (L) at residue 109. Self‐antigens were shown in bold letters. The residues 103L + 109L, 103L + 109F, 103V + 109F and 103M + 109L were highlighted in grey. The proteins carrying residues 103V + 109L that showed negative reactivity in AE2 column were highlighted in light green.

### 3.5 Subject 3 (S3): Identification of a Possible Continuous Linear Epitope Containing DEPs 107G ~ 109F

Residue 109 was recognised as DEP from the analysis of the SAB assays using the serum collected from S2 as described above. Of interest, in S3 serum, we identified positive reactivity to almost all HLA‐A SABs including HLA‐A*02:10, except for ‐A*02:01, ‐A*02:03, ‐A*02:05, ‐A*02:06, ‐A*02:07, ‐A*02:18 and ‐A*69:01, suggesting that the DEP 107G may determine in part the reactivity of this serum. A more complete understanding of this reactivity can be obtained when this substitution is paired in the same allele with the presence of 109F. This was the case because this serum also showed positive reactivity to the SAB HLA‐B*35:02 (Table S1C). This finding allowed us to propose the definition of the new Associated Antigen B‐3502; this novel Associated Antigen is rooted in the HLA‐B35 antigen. It can be proposed that a potentially shared epitope determined in part by DEPs 107G and 109F may explain some of the reactivity found in this serum.

## 4 Discussion

**FIGURE 5:**
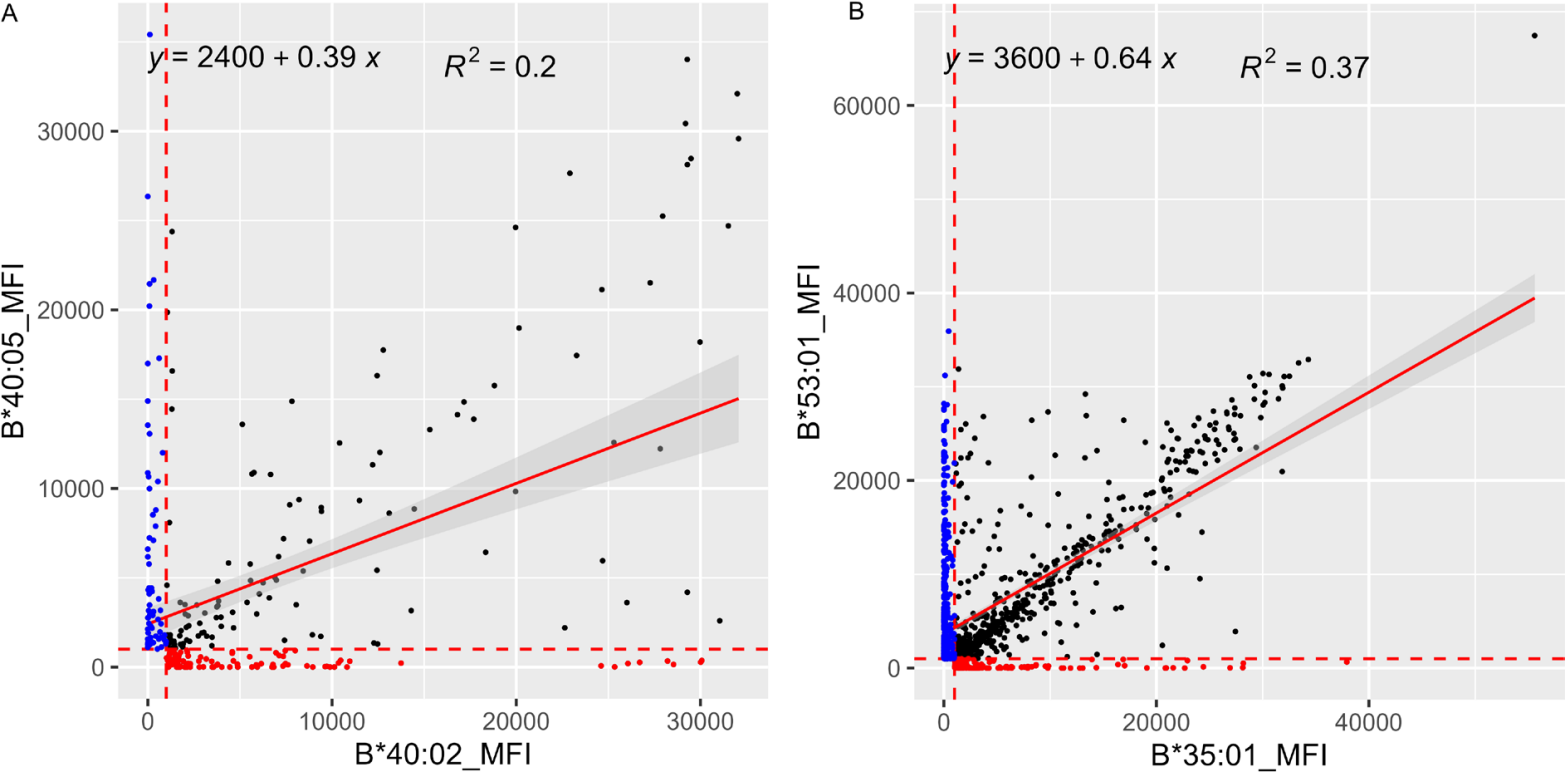
Scatter plots and linear regression analyses of the MFI values between the SABs HLA‐B*40:02 versus ‐B*40:05 and HLA‐B*35:01 versus ‐B*53:01. Scatter plots of MFI values are shown from SABs HLA‐B*40:02 versus ‐B*40:05 (A) and HLA‐B*35:01 versus ‐B*53:01 (B). The MFI values from the HLA‐B*40:02 and ‐B*35:01 are represented on the *X*‐axis, and HLA‐B*40:05 and ‐B*53:01 are on the *Y*‐axis. MFI values < 1000 on both SABs are omitted. The MFI value 1000 is shown as red dotted lines on the *X* and *Y* axes. The HLA‐B*40:02 and ‐B*35:01 specific positive reactivities (MFI > 1000) are shown in red dots, and the HLA‐B*40:05 and ‐B*53:01 specific positive reactivities (MFI > 1000) are shown in blue dots. The linear regression lines are represented in red. Both SABs HLA‐B*40:02/‐B*35:01 and ‐B*40:05/‐B*53:01 that are in the positive range (MFI > 1000) are shown with black dots. (A) The antigens B61 (HLA‐B*40:02) and B4005 (‐B*40:05) show very distinctive serological reactivity, and the single AA substitution at residue 163 is highly immunogenic. (B) The antigens B‐3501 (HLA‐B*35:01) and B53 (‐B*53:01) very distinctive serological reactivity due to the AA substitutions defining Bw4 and Bw6 specificities.

The aim of this study was to expand or confirm the criteria for the definition of serologic specificities corresponding to common HLA alleles in all world populations reported in the CIWD3.0 catalogue [17]. It is generally thought that the common alleles may have been positively selected locally because these may provide selected advantages and extend the gene pool of a given population. Many of the common alleles differ from other common alleles by a few nucleotide and amino acid differences that resulted from likely gene conversion events (e.g., HLA‐B*40:02/‐B*40:05, ‐B*35:01/‐B*53:01); in spite of these small amino differences, the regression analyses show distinct and dramatic serologic differences (Figure 5). It appears that the HLA allele diversity may have followed parsimonious processes involving point mutations, gene conversions involving short segmental exchanges and less often exon shuffling events [10, 18, 19, 20]; these events may generate alleles that encode for proteins that are serologically distinct. Paradoxically, some alleles that appear to have distant evolutionary origins, HLA‐B*15:03 (common in African populations) and HLA‐B*48:02 (found virtually only in Natives from South America), have similar serologic types (HLA‐B72 Associated Antigens B‐1503 and B‐4802) as being defined on the basis of specific amino acids that define serologic epitopes (Figure 3F). The protein HLA‐B*48:02 is a hybrid between HLA‐B*35:01 and HLA‐B*48:01 [20]. Similar examples of HLA‐B allele pairs that include alleles with likely distant evolutionary origin and are closely related in their serologic behaviour include B*51:02/B*53:01 (Figure 1), B*40:05/B*50:01 (Figure 3E). The precise serologic characterisation of all common HLA alleles in the world population would have been difficult at the time when no molecular typing methods were applied because only serologic methods were used for both HLA typing and the definition of the antigen diversity.

The present study was designed to assess the role of amino acid substitutions at residue 103 located in a connecting loop of HLA class I proteins in determining serologic epitopes. Introduction of the supplemental SABs in the routine clinical tests has provided the opportunity for testing additional proteins allowing for examining several additional antigen pairs differing at residue 103 and further refinement in the definition of the serological specificities. The anti‐HLA antibody adsorptions were performed with lymphocytes [13] or single allele recombinant HLA‐molecules immobilised on solid phases [14]. The eluates were tested by SP‐SAB assays or by flow cytometric cross‐matches with live lymphocytes to confirm the validity of DEP in HLA molecules in a native protein conformation.

**FIGURE 6:**
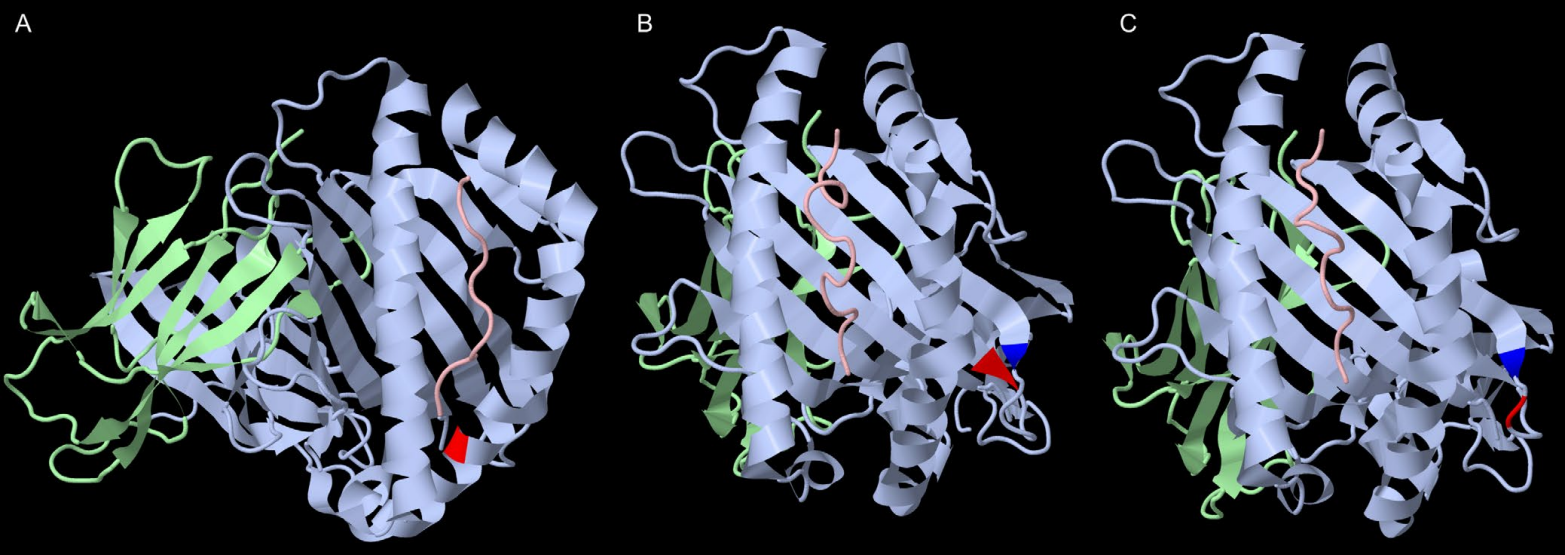
Residues 103, 107, 109 and 167 on HLA‐B molecules. (A) Residue 167 is shown in red on HLA‐B*44:02 molecule (https://doi.org/10.2210/pdb1M6O/pdb). The image is rotated for better visibility of residue 167. (B) Residues 103 and 109 are shown in red and blue, respectively, on HLA‐B*07:02 molecule (https://doi.org/10.2210/pdb6at5/pdb). (C) Residues 107 and 109 are shown in red and blue, respectively, on HLA‐B*35:01 molecule (https://doi.org/10.2210/pdb4LNR/pdb).

The presence of 167G or 167S appears to determine shared epitope(s) among proteins HLA‐A*01:01, ‐A*23:01, ‐A*24:02, ‐A*80:01, ‐B*15:12, ‐B*44:02, ‐B*44:03, ‐B*45:01, ‐B*50:02 and ‐B*82:01 (Table 2A); the closely related proteins bearing 167W do not carry these epitopes. Virtually identical observations were shown in a previous publication that included similar adsorption/elution experiments using white blood cells [13]. We speculate that cross‐reactivity among the positive proteins may result from the similar physicochemical characteristics of the amino acids Glycine and Serine replaced at residue 167. The proteins HLA‐B*50:02 and ‐B*50:01 differ only by the substitution S/W at residue 167; therefore, the reactivity of the eluate can only be ascribed to the presence of 167S in the positive cells. The substitutions 167G and 167S were previously described as being equivalent since the proteins HLA‐B*15:12 (167G) and HLA‐B*15:14 (167S) that differ at this residue were designated as bearing the same serologically defined antigen HLA‐B76 [10]; these proteins carry the negatively charged Aspartic acid (D) and Glutamic acid (E), respectively, at the contiguous residue 166. The residues 166D and 167G in HLA‐B*15:12 are unique among HLA‐B molecules and are characteristically found in HLA‐A*01:01, ‐A*23:01, ‐A*24:02 and ‐A*80:01; an inter‐locus gene conversion event between the HLA‐B*15:01 as a recipient allele and one of these *HLA‐A* alleles as sequence donor was suggested in the generation of the allele HLA‐B*15:12 [10]. The protein HLA‐B*15:14 differs from the HLA‐B*15:01 by the sole substitution of S for W at position 167; with the exception of HLA‐B*15:12 all common HLA‐B proteins carry the 166E. The DEP 167S is shared with alleles encoding the B12 antigens, that is, HLA‐B*44:02, HLA‐B*44:03 and HLA‐B*45:01, and is critical in forming the B12 epitope [21]. Serological cross‐reactivity between the HLA‐B76 and ‐B45 was previously explained by this shared residue 167S [22]. Similarly, a gene conversion between the allele HLA‐B*15:01 and one of the HLA‐B*44 or ‐B*45 alleles was suggested to result in the generation of the allele HLA‐B*15:14 [10]. Our results suggest that small size amino acids [Glycine (G) and Serine (S)] at residue 167 appear to show equivalent serologic behaviour while the antigens with presenting larger aromatic amino acid [Tryptophan (W)] residue displayed distinct serologic reactivity. Tryptophan's side chain consists of a six‐membered benzene ring fused to a five‐membered pyrrole ring, making it a large, aromatic, and relatively hydrophobic side chain with a unique structure among the 20 different amino acids that are commonly found in humans. The presence of the negatively charged amino acids D or E at residue 166 may result in a functionally conservative change. It is interesting to note that the antigen HLA‐A80 (HLA‐A*80:01) showed lower MFI in both the untreated and eluate S1 specimens, compared to the MFI of other SAB bearing DEP 167G/S. These differences may result from the presence of antibodies recognising at least two distinct sets of epitopes: one defined by the presence of DEP 167G/S while a second set may be defined by this DEP combined with other DEP(s) absent in HLA‐A80. A slightly different but not exclusive interpretation may result from differential antibody affinities for epitopes involving DEP 167G/S resulting from the presence of other DEP. The position of residue 167 is shown on HLA‐B*44:02 in Figure 6A.

Among the HLA‐B35 antigen, the most common proteins in Mexican populations include both HLA‐B*35:12 and ‐B*35:17 [17]. The definition of the B*35:12 as FULL serotype includes these two proteins that carry DEP 103V; in contrast, the common protein HLA‐B*35:01 and other common HLA‐B*35 proteins carry DEP 103L. The protein HLA‐B*35:12 differs from HLA‐B*35:01 by three amino acid substitutions at residues 103, 114 and 116 (Figure S2) while HLA‐B*35:17 differs from HLA‐B*35:01 by two amino acid substitutions at residues 97 and 103 (Figure S3). It has been speculated that both alleles were generated on the American continent by two different gene conversion events involving segmental exchanges of different lengths involving the Native American founder alleles HLA‐B*35:01 as a backbone recipient allele and B*40:02 as a donor of the segments introduced in the backbone allele [18]. The proteins HLA‐B*35:12 and ‐B*35:17 differ by three amino acid replacements: 97 (pockets C and E), 114 (pockets D and E) and 116 (pocket F); these are located at the bottom of the groove (Figure S4). These structural differences are likely to result in differential peptide binding profiles (or immunopeptidomes) that suggest that the mismatch in these alleles may result in an immunogenic HLA mismatch eliciting T‐cell allo‐reactivity. As identified in the previous validation study, the proteins that differ only at non‐DEP residues display high correlation in the regression analyses and close serologic reactivity [6]. Residues 97, 114 and 116 have been defined as being non‐DEP and were excluded from the serologic definition of serologic antigens in the regression analyses of other pairwise comparisons. It can be extrapolated that the mismatch in the proteins HLA‐B*35:12 and ‐B*35:17 that show differences only at these non‐DEP residues may elicit low or no B‐cell allo‐reactivity. Although the crystallography of these proteins and many other HLA proteins has not been determined, we have proposed that alleles having identical replacements at DEP residues belong to the same FULL serotype and it is assumed that they have similar serologic behaviour. The crystallographic studies of other closely related proteins show similar spatial configurations [23]; it cannot be ruled out that future and more detailed studies may detect distinguishing serologic differences and the definition of further serologic splitting. On a similar aspect, alleles having distinct immunopeptidomes (because of differences in peptide binding pockets) may display different sets of peptides that in turn may determine some peptide‐dependent serologic epitopes as those that have been recently identified [24]. The accuracy of the evaluation of the presence of antibodies directed against epitopes defined by the bound peptides and their clinical significance may be difficult since the expression of those epitopes will depend on the abundance in the intracellular pool of peptides that may vary significantly in different cell types and their activation status and may be over‐ or under‐represented in vitro prepared recombinant molecules included in the SAB panels. Under the assumptions indicated above, SAB panels including the majority of common HLA serologically defined at the FULL status may allow for selection with higher accuracy of equivalent SAB as surrogate antigens for the assessment of DSA. For example, the SAB HLA‐B*35:12 that is included in the expanded panel of SAB can serve as a surrogate for accurately tracking DSA against HLA‐B*35:17.

S2 Serum was adsorbed with lymphocytes carrying HLA‐B*35:12+HLA‐B*35:17 (AE2); in addition, the anti HLA‐B*57:01 antibodies were also enriched using the SAB coated with HLA‐B*57:01 separately. Unexpectedly, it was observed that the SABs HLA‐A*32:01, ‐A*74:01, ‐C*03:02, ‐C*03:03 and ‐C*03:04 also showed strong reactivity using the different eluates. Residue 109 was not considered initially as a DEP in previous studies because the antigens HLA‐A32 and ‐A74 could be explained without the consideration of residue 109 [5]. The results of these assays indicate that both HLA‐A32 and A74 share epitopes present in the adsorbing HLA molecules. The crystal structure of HLA class I molecules shows that residues 103 and 109 are in external connecting loops and are in physical vicinity [25, 26, 27]. This multi‐locus cross‐reactivity is most likely explained by the fact that allo‐antibodies present in this serum recognise one or more putatively defined discontinuous epitope(s) determined by the simultaneous presence of residues 103V and 109L in the same molecule (Figure 6B). To our knowledge, this is the first report describing the putative discontinuous shared epitope including DEPs 103V + 109L that is responsible for cross‐reactivity and defines the inter‐locus epitope corresponding to antigens encoded in three different classical class I loci (HLA‐A, ‐B and ‐C). Interestingly, residues 103L and 109F are listed in the eplet registry. The alternative replacements 103V and 109L are not recognised as eplets because they are conserved in all common HLA‐A and HLA‐C antigens, respectively [11]. The combination of both residues appears to determine the inter‐locus epitope described in the present study.

The complexity of the possible epitopes detected in the S1‐AE2 eluate (obtained after adsorption with lymphocytes bearing the alleles HLA‐B*35:12 and ‐B*35:17 that carry 103V + 109L) illustrates the limitations in applying single eplet or single residue analysis for making predictions of serum reactivity with (donor) HLA antigens not represented in the SAB panels. Furthermore, the precise identification and characterisation of antibodies recognising different epitopes in this serum would require series and sequential adsorption and elution experiments. In order to address the limitations in VXM for many patients, a practical decision in the design of an SAB panel would be to expand it by including the majority or all the antigens corresponding to common alleles found in all world populations as listed in the CWD2.0 and CIWD3.0 catalogue [17, 28].

The Associated Antigen HLA‐A210 was initially described by its negative reactivity with some sera and monoclonal antibodies reactive with the vast majority of cells bearing the HLA‐A2 antigen in the 9th International Histocompatibility Workshop (IHW) [29]; this antigen was defined at the 11th IHWS by its negative reactivity with mAb reactive with most HLA‐A2 cells and HLA‐A69 [22, 30]. In the present study, a reverse case was found that a serum collected from a subject S3 who is homozygous for HLA‐A*02:01 showed positive reactivity to the SAB HLA‐A*02:10, and was negative to the SABs HLA‐A*02:01, ‐A*02:03, ‐A*02:05, ‐A*02:06, ‐A*02:07, ‐A*02:18 and ‐A*69:01. Interestingly, this serum displayed reactivity to the SAB HLA‐B*35:02 with a significantly high MFI value and negative or low MFI reactivity to the SABs bearing HLA‐B*35:01, ‐B*35:03 and ‐B*35:08 (Table S2). Based on these observations, we proposed the designation of B‐3502 as an Associated Antigen. It is possible that S3 serum may detect one or more epitopes present in HLA proteins bearing DEPs 107G and 109F (Figure 6C). These DEPs are simultaneously present in HLA‐B*35:02 and most HLA‐A common proteins excluding HLA‐A32 and HLA‐A74 antigens as well as most of the common proteins bearing HLA‐A2 serologic specificity.

Structural or functional dimorphisms appear to determine additional serologic heterogeneity in HLA class II proteins as well. The AA dimorphisms with the same charge [Arginine (R) vs. Lysine (K)] at residue 69 of HLA‐DPB1 and at residue 71R/K of HLA‐DRB1/3/4/5 did not result in a significantly different serological behaviour, and the antigens bearing these differences correspond to the same serological specificity [6]. However, these rules cannot be generalised to substitutions in other AA positions. A recent publication identified that the dimorphism (Arginine vs. Lysine) at residue 96 of HLA‐DPB1 determines additional serologic variation [16]. It is interesting to note that residue 96 of HLA‐DPB1 is in a more proximal location to the cell membrane. In a previous report, all DEPs selected for the definition of serological specificities were located on the distal membrane domain [5]. Initially, we did not propose different antigens considering the variation at residue 96 of DPB1 due to a lack of clear scientific evidence supporting that variations at this position determine serologically defined allo‐epitope. Recently, we re‐evaluated previous data and examined new tests, including the supplemental SAB panels that allowed us to confirm the result of the recent report [16]. The new analyses allowed us to identify sera that distinguish differences in pairs of SAB, including HLA‐DPB1 proteins that differ only at residue 96 and carry other identical HLA‐DPB1 DEP paired with the same DPA1 protein (e.g., HLA‐DPB1*06:01/17:01 and ‐DPB1*18:01/28:01, Table S3). The results of our analyses and those shown in the previous publication led us to propose the definition of new serological specificities corresponding to HLA‐DPB1 antigens. The inclusion of residue 96 in the HATS v3.0 logic for the definition of HLA‐DPB1 serotypes will allow for a more accurate assessment of anti‐HLA‐DP antibody reactivity.

The understanding and listing of the newly described serotypes and Associated Antigens can be used in the routine clinical histocompatibility practice [17, 28]. Variations of reagent quality may not be readily identified when a new batch of reagents is being introduced into clinical practice. These variations could be assessed in the evaluation SP‐SAB assays; the reactivity of groups of HLA SAB corresponding to alleles that belong to the same serotype are expected to show MFI in similar range (e.g., Associated Antigen B‐4402 SAB includes both HLA‐B*44:02 and HLA‐B*44:03). When contrasting MFI values for the representative SAB of the group are detected for a given patient's serum, the MFI discordance may correspond to the spurious reactivity associated with the presence of some denatured HLA molecules coated on SP‐SAB. It has been noted spurious reactivity corresponding to the SAB for the first allele listed in the groups of SAB corresponding as follows: (1) HLA‐A*02:07/‐A*02:01/‐A*02:06, Associated Antigen A‐0201; (2) HLA‐B*44:02/‐B*44:03, Associated Antigen B‐4402; (3) HLA‐B*13:02/‐B*13:01, Antigen B13; (4) A*11:02/A*11:01, Antigen A11. These spurious reactivities in SP‐SAB assays were confirmed as such by performing cell based FXM with informative with lymphocytes from donors with informative HLA‐genotypes that were invariably negative [6].

In summary, the application of serum adsorption/elution using lymphocytes and SAB coated with a specific antigen provides a powerful additional tool for the confirmation and identification of novel serological specificities; these methods also allow for identification and elucidation of the role of DEPs contributing to less known cross‐reactivities. The residues 167G and 167S are confirmed to be serologically equivalent; they contribute to some cross‐reactivities between the antigens in HLA‐A (A1, A‐2301, A‐2402 and A80) and the antigens in HLA‐B (B44, B45, B76 and B82). The DEPs 103V + 109L combined were confirmed to contribute to the cross‐reactivities among HLA‐A, ‐B and HLA‐C antigens. Taking into consideration DEPs 103, 18 common proteins that were catalogued previously in the SEROTYPE status can now be defined in the 12 FULL antigen category. In addition, the combination of DEPs 107G~109L in many of the HLA‐A antigens supports proposing a newly recognised Associated Antigen B‐3502. The current work documents that the AA substitutions at residues 103 and 109 of HLA class I can be included in the criteria for defining Associated Antigens.

In conclusion, we included 13 HLA‐B and 5 HLA‐DPB1 newly confirmed antigens listed in Table 5 to be approved by the WHO Nomenclature Committee for Factors of the HLA System. The updated reference table is shown in Table S4. Considering the 198 common HLA‐B proteins in all world populations reported in the CIWD3.0 catalogue, [17], 194 proteins are included in the FULL antigen category and only 4 proteins remain in the SEROTYPE category (Table S5).

**TABLE 5:** Newly proposed associated antigens.

	Proposed associated antigens				WHO recognised antigen	
ID	Proposed	DEP1	Previous	DEP2	Split	Broad
1	B‐0712	103L	B‐0702	103V		B7
2	B‐4410	103L	B‐4402	103V	B44	B12
3	B‐1520	103L	B‐1501	103V	B62	B15
4	B‐5504	103V	B‐5501	103L	B55	B22
5	B‐5603	103V	B‐5601	103L	B56	B22
6	B‐2714	103L	B‐2705	103V		B27
7	B‐3512	103V	B‐3501	103L		B35
8	B‐3516	103V	B‐3510	103L		
9	B‐3531	103V	B‐3515	103L		
10	B‐4004	103L	B‐4002	103V	B61	B40
11	B‐4802	103L	B‐1503	103V	B72	B70
12	B‐3521	103L	B‐7801	103V		B78
13	B‐3502	109F	B‐3501	109L		B35
14	DP‐15	96K	DP‐401	96R		
15	DP‐17	96R	DP‐06	96K		
16	DP‐18	96K	DP‐0402	96R		
17	DP‐30	96R	DP‐13	96K		
18	DP‐31	96R	DP‐01	96K		

*Note:* ‘Proposed’ column shows the newly proposed antigens in this study. ‘Previous’ column shows the antigens that were reported previously. ‘DEP1’ and ‘DEP2’ columns show DEPs that distinguish the proposed antigens from the previously reported antigens. ‘Split’ and ‘Broad’ columns show the currently WHO recognised antigens. Three residue positions were taken into consideration for the newly proposed antigens: residues 103 and 109 for HLA‐B and residue 96 for HLA‐DPB1.

## Author Contributions

This study was designed by K.O. and M.A.F.V. J.S. and K.Y. performed adsorption and elution. K.O. and M.A.F.V. were also involved in data interpretation and drafting the manuscript. K.O. updated HATS. S.G.E.M. contributed to reviewing HLA nomenclature including antigens.

## Conflicts of Interest

The authors declare no conflicts of interest.

## Supporting information


**Figure S1:** SAB assays using three sera for S2.
**Figure S2:** Protein HLA‐B*35:12 differs from HLA‐B*35:01 by three amino acid substitutions at residues 103, 114 and 116.
**Figure S3:** Protein HLA‐B*35:17 differs from HLA‐B*35:01 by two amino acid substitutions at residues 97 and 103.
**Figure S4:** Protein HLA‐B*35:17 differs from HLA‐B*35:12 by three amino acid substitutions at residues 97, 114 and 116.

**Table S1:** Correlation analyses among HLA‐A SABs.

**Table S2:** Recognition of the antigen B‐3502.

**Table S3:** Newly proposed HLA‐DPB1 antigens DP‐17, DP‐18 and DP‐30.

**Table S4:** Proposed associated antigens, WHO recognised antigens and their residues determining epitope (DEP) for HLA‐DR.

**Table S5:** Status of HLA serologic definition.

## Data Availability

The data that support the findings of this study are available on request from the corresponding author. The data are not publicly available due to privacy or ethical restrictions.
