# Supplementary material for: Replacements at Structural or Functional Dimorphisms 103, 109 and 167 Distinguish HLA Class I Serologically Defined Antigens
Source: HLA. 2025 Sep 13;106(3):e70387. doi: 10.1111/tan.70387 (PMC12432678; doi:10.1111/tan.70387)
Supplement: Supplementary file 1 — Figure S1: SAB assays using three sera for S2. Figure S2: Protein HLA‐B*35:12 differs from HLA‐B*35:01 by three amino acid substitutions at residues 103, 114 and 116. Figure S3: Protein HLA‐B*35:17 differs from HLA‐B*35:01 by two amino acid substitutions at residues 97 and 103. Figure S4: Protein HLA‐B*35:17 differs from HLA‐B*35:12 by three amino acid substitutions at residues 97, 114 and 116. [file TAN-106-e70387-s002.docx]

Supplemental Figure 1: SAB assays using three sera for S2


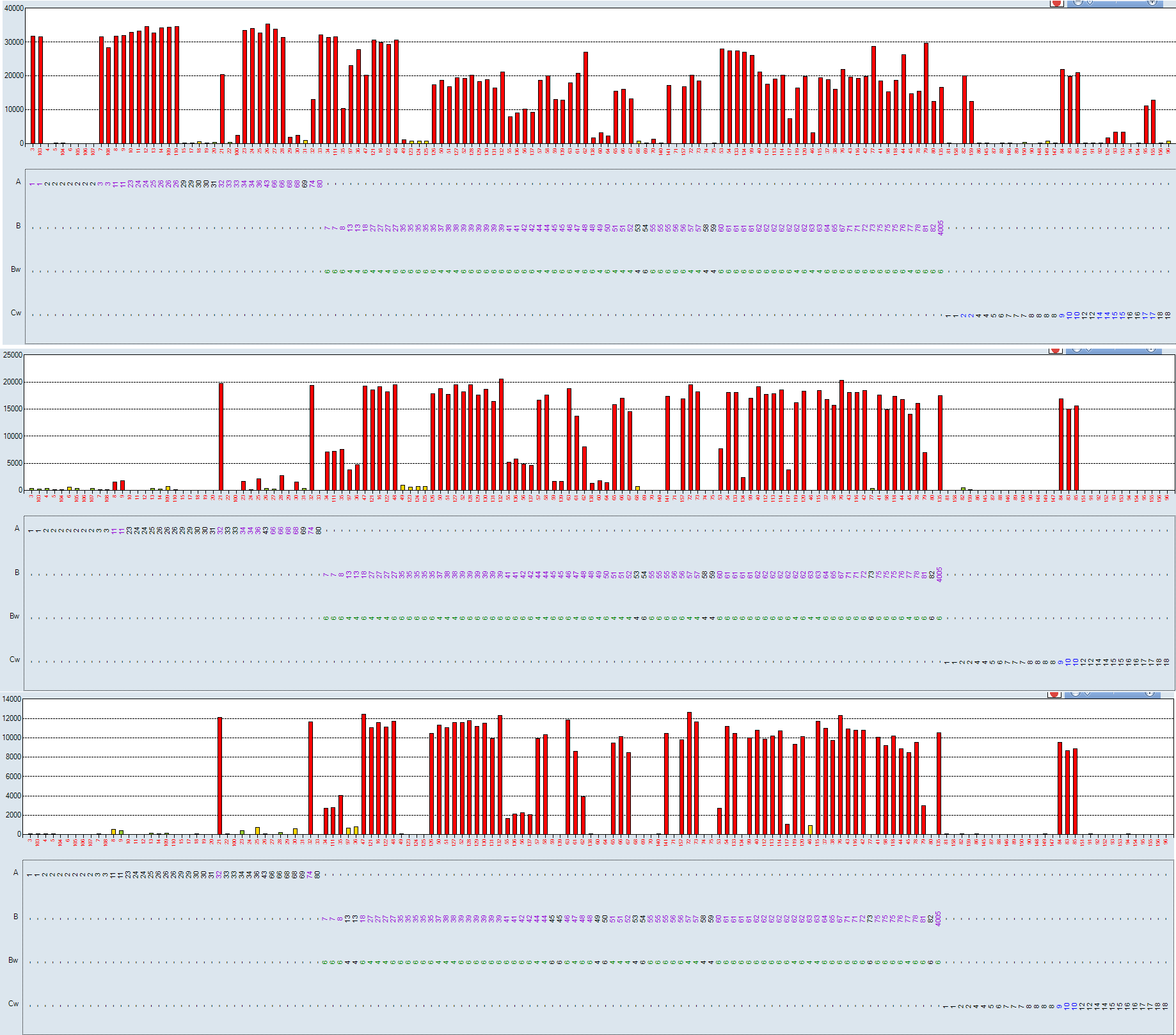


SAB assay results are displayed using Fusion software. Top: Results using untreated serum from S2 are shown. Middle: Results using eluate (S2-AE2) that is adsorbed and eluted using cells expressing HLA-B*35:12+HLA-B*35:17 are shown (AE2 in Table 1). Bottom: Results using eluate (S2-AE3) that is adsorbed and eluted using MagSort SAB coated with HLA-B*57:01 are shown (AE3 in Table 1). Nearly identical reactive patterns are observed from these eluates with two different antigens [B-3512 (middle) and B57 (bottom)]. All HLA-B SABs containing DEP 103V showed positive reactivity, while the HLA-B SABs containing 103L were all negative. In addition to the reactivity related to the HLA-B SABs containing DEP 103V, the sera showed strong reactivity to the SABs HLA-A*32:01, HLA-A*74:01 and HLA-C*03:02, HLA-C*03:03 and HLA-C*03:04.

Supplemental Figure 2: Protein HLA-B*35:12 differs from HLA-B*35:01 by three amino acid substitutions at residues 103, 114 and 116


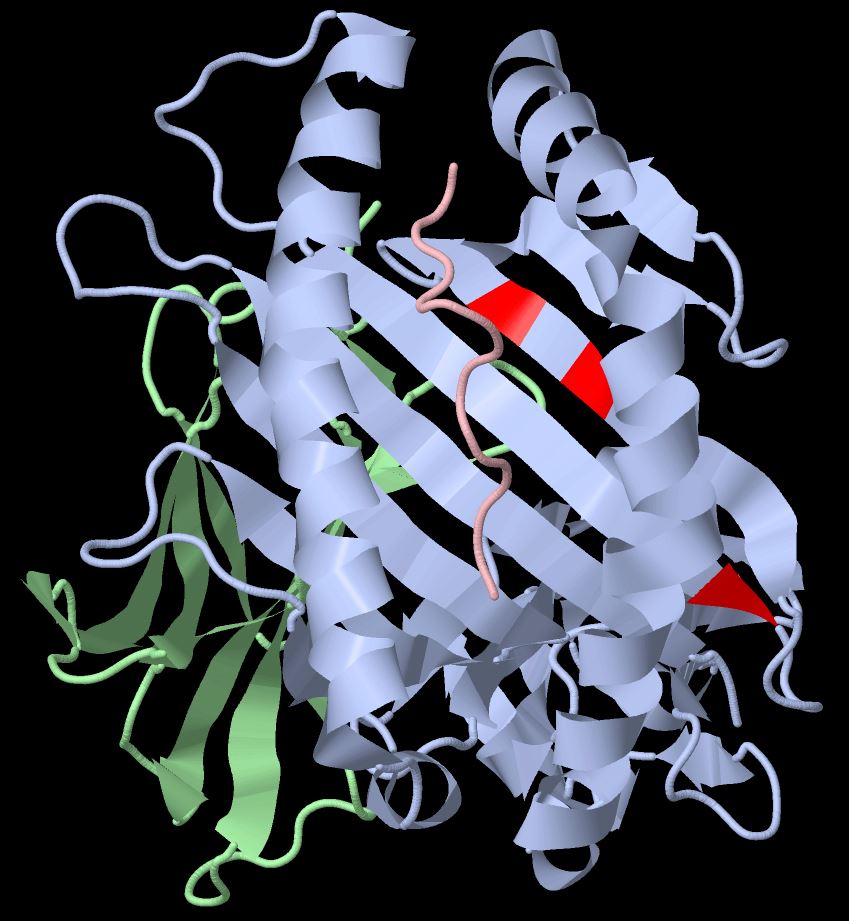


Residues 103, 114 and 116 are shown in red on HLA-B*35:01 molecule (https://doi.org/10.2210/pdb4LNR/pdb).

Supplemental Figure 3: Protein HLA-B*35:17 differs from HLA-B*35:01 by two amino acid substitutions at residues 97 and 103


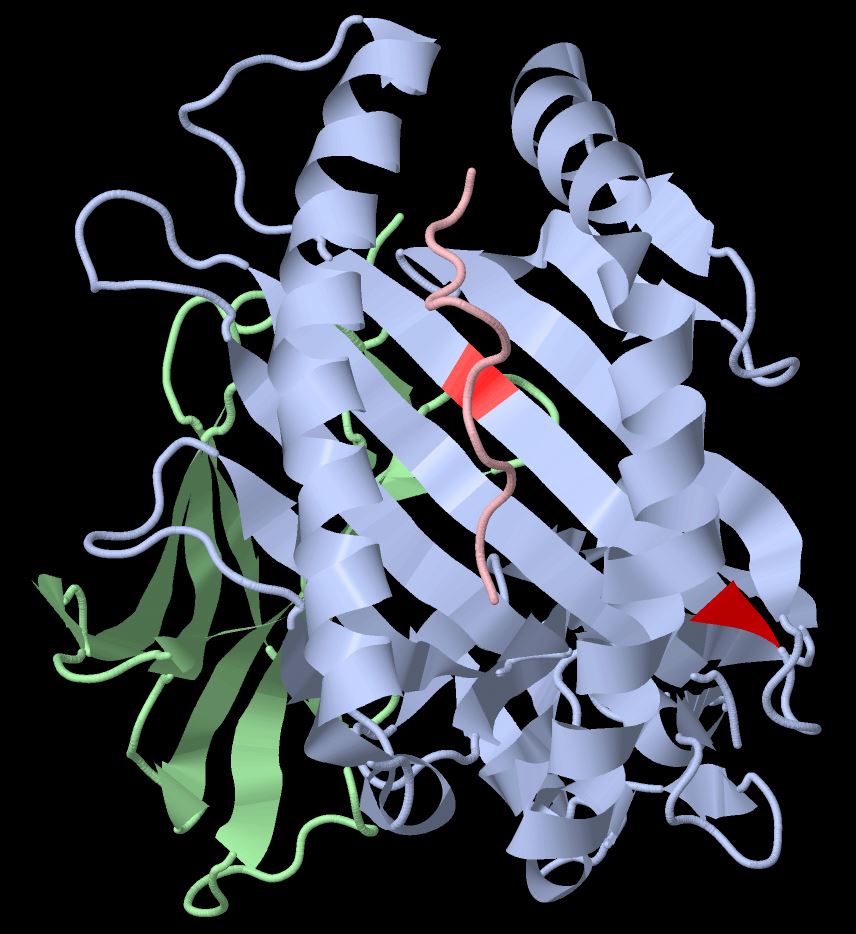


Residues 97 and 103 are shown in red on HLA-B*35:01 molecule (https://doi.org/10.2210/pdb4LNR/pdb).

Supplemental Figure 4: Protein HLA-B*35:17 differs from HLA-B*35:12 by three amino acid substitutions at residues 97, 114 and 116


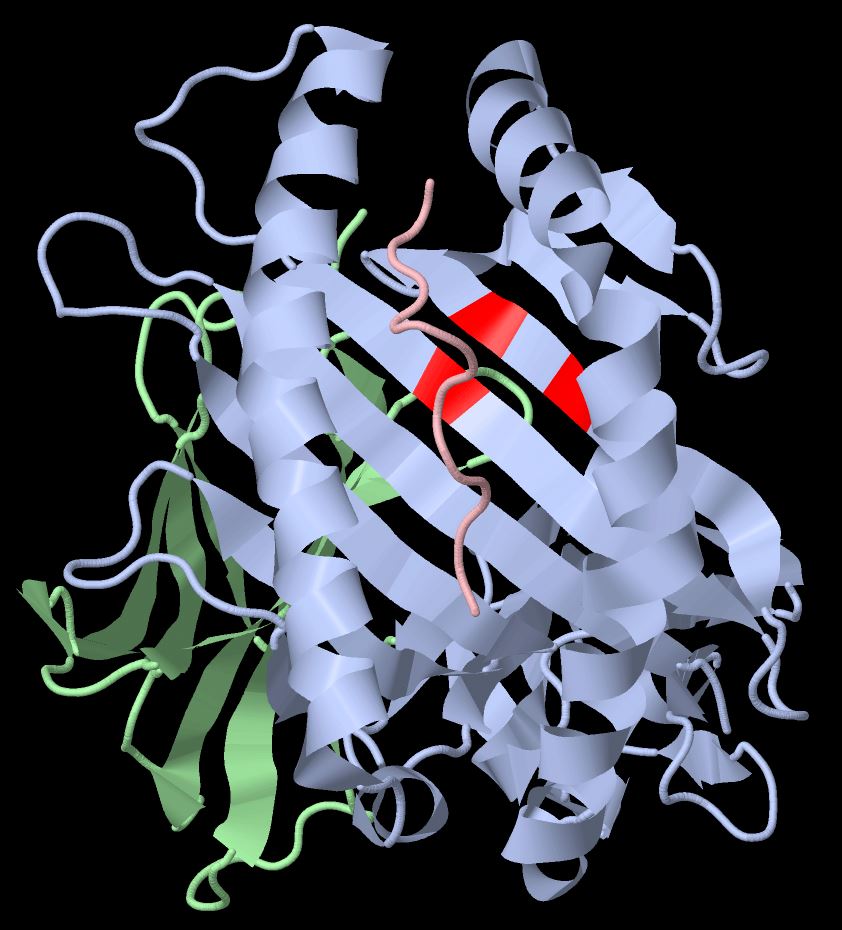


Residues 97, 114 and 116 are shown in red on HLA-B*35:01 molecule (https://doi.org/10.2210/pdb4LNR/pdb).
