# Supplementary material for: Replacements at Structural or Functional Dimorphisms 103, 109 and 167 Distinguish HLA Class I Serologically Defined Antigens
Source: HLA. 2025 Sep 13;106(3):e70387. doi: 10.1111/tan.70387 (PMC12432678; doi:10.1111/tan.70387)
Supplement: Supplementary file 3 — Table S2: Recognition of the antigen B‐3502. [file TAN-106-e70387-s003.docx]

Supplemental Table 2: Recognition of the antigen B-3502

| Antigen | allele | MFI | 107 | 109 |
| --- | --- | --- | --- | --- |
| A1 | A*01:01 | 28463 | G | F |
| A1 | A*01:02 | 28901 | G | F |
| A2 | A*02:01 | 161 | W | F |
| A2 | A*02:03 | 62 | W | F |
| A2 | A*02:05 | 278 | W | F |
| A2 | A*02:06 | 104 | W | F |
| A2 | A*02:07 | 241 | W | F |
| A2 | A*02:10 | 9226 | G | F |
| A2 | A*02:18 | 31 | W | F |
| A3 | A*03:01 | 27551 | G | F |
| A3 | A*03:02 | 27702 | G | F |
| A11 | A*11:01 | 25960 | G | F |
| A11 | A*11:02 | 24019 | G | F |
| A23 | A*23:01 | 22307 | G | F |
| A24 | A*24:02 | 27459 | G | F |
| A24 | A*24:03 | 29994 | G | F |
| A25 | A*25:01 | 28591 | G | F |
| A26 | A*26:01 | 28189 | G | F |
| A26 | A*26:02 | 27781 | G | F |
| A26 | A*26:03 | 28545 | G | F |
| A29 | A*29:01 | 25436 | G | F |
| A29 | A*29:02 | 26543 | G | F |
| A30 | A*30:01 | 27147 | G | F |
| A30 | A*30:02 | 27220 | G | F |
| A31 | A*31:01 | 26523 | G | F |
| A32 | A*32:01 | 19102 | G | L |
| A33 | A*33:01 | 24159 | G | F |
| A33 | A*33:03 | 26724 | G | F |
| A34 | A*34:01 | 21583 | G | F |
| A34 | A*34:02 | 26214 | G | F |
| A36 | A*36:01 | 28878 | G | F |
| A43 | A*43:01 | 27478 | G | F |
| A66 | A*66:01 | 26939 | G | F |
| A66 | A*66:02 | 21133 | G | F |
| A68 | A*68:01 | 7391 | G | F |
| A68 | A*68:02 | 5964 | G | F |
| A69 | A*69:01 | 363 | W | F |
| A74 | A*74:01 | 28639 | G | L |
| A80 | A*80:01 | 28662 | G | F |
| B35 | B*35:01 | 716 | G | L |
| B35 | B*35:02 | 10031 | G | F |
| B35 | B*35:03 | 327 | G | L |
| B35 | B*35:08 | 1086 | G | L |
| B35 | B*35:12 | 5176 | G | L |
| B57 | B*57:01 | 15883 | G | L |
| B57 | B*57:03 | 14429 | G | L |
| B58 | B*58:01 | 8451 | G | L |

S3 Serum showed reactivity to almost all HLA-A SABs including HLA-A*02:10 except for -A*02:01, -A*02:03, -A*02:05, -A*02:06, -A*02:07, -A*02:18 and -A*69:01. This serum also showed positive reactivity to the SAB HLA-B*35:02.
