# Supplementary material for: Replacements at Structural or Functional Dimorphisms 103, 109 and 167 Distinguish HLA Class I Serologically Defined Antigens
Source: HLA. 2025 Sep 13;106(3):e70387. doi: 10.1111/tan.70387 (PMC12432678; doi:10.1111/tan.70387)
Supplement: Supplementary file 4 — Table S3: Newly proposed HLA‐DPB1 antigens DP‐17, DP‐18 and DP‐30. [file TAN-106-e70387-s005.docx]

Supplemental Table 3: Newly proposed HLA-DPB1 antigens DP-17, DP-18 and DP-30

| Serum | Previous | Updated | SAB | 96 | MFI |
| --- | --- | --- | --- | --- | --- |
| S4 | DP-13 | DP-13 | DPA1*02:01~DPB1*13:01 | K | 0 |
|  |  | DP-30 | DPA1*02:01~DPB1*30:01 | R | 21596 |
|  | DP-06 | DP-06 | DPA1*02:01~DPB1*06:01 | K | 0 |
|  |  |  | DPA1*02:01~DPB1*09:01 | K | 0 |
|  |  | DP-17 | DPA1*02:01~DPB1*17:01 | R | 20601 |
| S5 | DP-0402 | DP-0402 | DPA1*01:03~DPB1*04:02 | R | 0 |
|  |  |  | DPA1*01:03~DPB1*28:01 | R | 234 |
|  |  | DP-18 | DPA1*01:04~DPB1*18:01 | K | 10159 |
|  |  |  | DPA1*01:05~DPB1*18:01 | K | 9066 |

“Serum” column shows serum ID. “Previous” column shows previously defined antigens without taking residue 96 into account. “Updated” column shows updated antigens that were defined as two distinct antigens based on the variations at residue 96.
